# Revealing influencing factors on global waste distribution via deep-learning based dumpsite detection from satellite imagery

**DOI:** 10.1038/s41467-023-37136-1

**Published:** 2023-03-15

**Authors:** Xian Sun, Dongshuo Yin, Fei Qin, Hongfeng Yu, Wanxuan Lu, Fanglong Yao, Qibin He, Xingliang Huang, Zhiyuan Yan, Peijin Wang, Chubo Deng, Nayu Liu, Yiran Yang, Wei Liang, Ruiping Wang, Cheng Wang, Naoto Yokoya, Ronny Hänsch, Kun Fu

**Affiliations:** 1 Aerospace Information Research Institute, Chinese Academy of Sciences, 100190 Beijing, China; 2 School of Electronic, Electrical and Communication Engineering, University of Chinese Academy of Sciences, 100190 Beijing, China; 3 Key Laboratory of Network Information System Technology (NIST), Aerospace Information Research Institute, Chinese Academy of Sciences, 100190 Beijing, China; 4 Institute of Computing Technology, Chinese Academy of Sciences, 100190 Beijing, China; 5 Fujian Key Laboratory of Sensing and Computing for Smart Cities, School of Information Science and Engineering, Xiamen University, 361005 Xiamen, China; 6 Fujian Collaborative Innovation Center for Big Data Applications in Governments, 350003 Fuzhou, China; 7 RIKEN Center for Advanced Intelligence Project, RIKEN, Tokyo, 103-0027 Japan; 8 Department of Complexity Science and Engineering, The University of Tokyo, Tokyo, 113-8654 Japan; 9 German Aerospace Center (DLR), 82234 Weßling, Germany

**Keywords:** Environmental economics, Sustainability, Environmental impact, Environmental impact

## Abstract

With the advancement of global civilisation, monitoring and managing dumpsites have become essential parts of environmental governance in various countries. Dumpsite locations are difficult to obtain in a timely manner by local government agencies and environmental groups. The World Bank shows that governments need to spend massive labour and economic costs to collect illegal dumpsites to implement management. Here we show that applying novel deep convolutional networks to high-resolution satellite images can provide an effective, efficient, and low-cost method to detect dumpsites. In sampled areas of 28 cities around the world, our model detects nearly 1000 dumpsites that appeared around 2021. This approach reduces the investigation time by more than 96.8% compared with the manual method. With this novel and powerful methodology, it is now capable of analysing the relationship between dumpsites and various social attributes on a global scale, temporally and spatially.

## Abstract (web summary)

Dumpsites are hard to locate globally. Here the authors apply deep networks to satellite images to provide an effective and low-cost way to detect dumpsites with the new method saving more than 96.8% of the manual time with a strong sensitivity to dumpsites.

## Introduction

With the global surge in waste production, management and discharge of dumpsites are receiving more attention than ever before. At the same time, carbon neutrality has been a goal of many governments over the years, with many countries already committed to achieving zero emissions by mid-century^1^. Greenhouse gas emissions from post-consumer waste account for 3% of global man-made greenhouse gas and 18% of the global anthropogenic CH_4_ emissions^2^. As the World Bank noted in 2018, “the world is on a trajectory where waste generation will drastically outpace population growth by more than double by 2050”^3^. Due to the rapid growth of global waste production, the supervision of both legal and illegal dumpsites is significant to global environmental governance. In fact, those produced waste will initially pile up into dumpsites, including large dumpsites and some small illegal dumps. Large dumpsites can result in the formation and spread of infectious diseases^4–7^, which may endanger the lives of scavengers^8–10^, prey hyenas^11^, bears^12,13^, birds^14,15^ living around. Some scholars also hold the opinion that targeted monitoring and management of large dumpsites could better increase the income level of scavengers around dumpsites^16^ and enhance the efficiency of waste recycling^17^. For small ones, World Bank pointed out that “unregulated or illegal dumpsites serve about 4 billion people and hold over 40% of the world’s waste”^18^. Scholars claim that illegal dumpsites are caused by illegal dumping by nearby residents^3^, making it difficult to track their location on a regular basis. Dumpsite information can positively affect research into human behaviour, geoscience, environmental protection, and more.

The primary task of dumpsite monitoring is to regularly confirm their locations, which the environmental department often does at enormous labour cost^19^. Globally 33% of waste is openly dumped, while this percentage in upper-middle and low-income countries is 30% and 93%, respectively^3^. Johannesburg collects 4500 tonnes of new illegally dumped waste every week and produces 1.56 million tonnes of waste every year^20^. It is challenging to manually locate small illegal dumpsites following classical method. As a result, these illegal dumpsites are usually reported by residents or discovered by the government after accumulating a certain volume. In addition, the peculiar smell and unhealthiness of dumpsites are also unfriendly to information collectors, so it is almost impossible to locate dumpsites on a global scale using manual methods. In fact, satellite imaging technology has become a powerful basis for earth observation in the last decade. Several works aim to use satellite images to locate dumpsites with manual and semi-manual methods^21–23^. Limited by the inflexibility of traditional algorithms and the low resolution of previous satellite images, labour costs are still very high in early attempts. Thus, there is an urgent need for an automated method with superior performance to apply advanced earth observation technology to dumpsite detection. Scholars employ Unmanned Aerial Vehicles (UAVs)^24^ to detect dumpsites with promising results, yet UAV is still inefficient for rapid detection on a global scale. Several works combine deep learning and earth observation for dumpsite detection, where data is collected locally in regions of a certain country, including Shanghai^25^, Johannesburg^20^, and cities in Italy^26,27^. Recent literature^28^ annotates major landfills at the pixel level in several countries and implements semantic segmentation with specific models, but only 13 mutually independent landfills are included. All the above remarkable works do not include publicly available dumpsite datasets. BigEarthNet^29^ contains a small number of unclassified dumpsites but is more commonly used for general land use classification^28^. Overall, existing literature has not publicly released specific global datasets for classified dumpsites, making it challenging for academics and institutions wishing to investigate further.

**Fig. 1:**
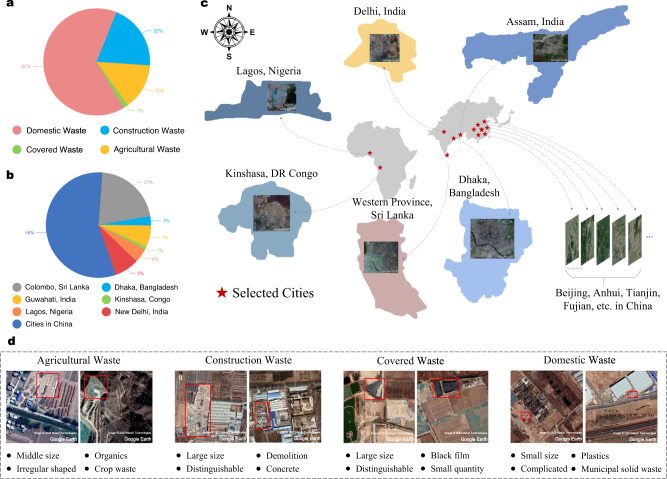
Basic information of dumpsite dataset and typical examples of four categories. **a** The proportion of the four categories. **b** Quantity distribution of dumpsite samples in different countries. (The number of dumpsites in China is relatively large since data in China is easier to obtain in this work, and our validation results show that the proposed method has the generalisability to detect dumpsites globally.) **c** Geographic location of all selected cities in our dataset for training and verification. **d** Typical examples and characteristics of the four types of dumpsites.

In order to assist academics and government environmental authorities in their studies of dumpsites globally, we adopt deep learning to reduce labour costs and the difficulties of locating dumpsites. Deep learning has been proven in many fields to achieve satisfactory results with automatic mechanisms, including computer vision^30^, remote sensing^31^, agriculture^32^, medicine^33^ and so on. To better apply deep learning to dumpsite detection, we carry out this work in terms of data and model. After long-term field research (see supplementary materials), we first construct a global dumpsite dataset (including numerous illegal dumps and a few regulated landfills) by manually labelling about 2500 dumpsites in nearly 4800 square kilometres of satellite images with high resolutions from 0.3 m to 1 m per pixel worldwide. We select areas of several representative cities in the world with larger populations and lower-ranking of environmental performance with the help of the world city population rankings in the 2016 World Cities Report^34^, the Global National Environmental Performance Index (EPI)^35^ rankings released by Yale University in 2020, and extensive literature and online resources. Figure 1b, c show the geographical distribution of selected cities and the proportion of dumpsites in each country. More importantly, we classify dumpsites into domestic waste, construction waste, agricultural waste, and covered waste without exception based on Fig. 1d and label their categories in the dataset. Agricultural waste, construction waste and domestic waste are common and well understood, which are made up of crops, construction debris and residential waste respectively and differ considerably in how they are disposed of and their appearance. Another less common class is the covered waste, which is covered with dark films (e.g., high density polyethene (HDPE) films) after being disposed of by professional waste management. Recent literature suggests that films can significantly reduce the concentration of volatile compounds (VOCs) and inhibit the production of waste permeate in the vicinity^36–38^, thereby reducing the health risks to nearby residents and the soil. Covered waste is vital for waste management, so we include it as a separate category and detect this class with advanced imbalance data countermeasures (see “Long-tailed distribution problem” in the supplementary materials for similar examples of data imbalance). It is worth noting that some other objects, including covered farming tools or construction equipment, may have similar outlooking of covered waste, e.g. with HDPE films. However, these objects are usually small in size and can be easily distinguished in the deep feature space. We have verified all the covered wastes on a case-by-case basis to ensure authenticity. To the best of our knowledge, previous studies^20–29^ do not include a case for fine-grained classification of global dumpsites from the perspective of satellite imagery which may help the government and related scholars conduct more fine-grained dumpsite supervision and research. Figure 1d illustrates the appearance of four types of dumpsites.

Considering the characteristics of the dumpsite and the limitations of the current typical deep learning model detailed in the subsequent Model section, we propose a novel deep model for dumpsite detection named BCA-Net. Experimental results indicate that our model can detect more than 98% of dumpsites, and the supplementary materials also demonstrate that our model outperforms other existing models in the task of dumpsite detection. With this powerful method, we are now able to analyse the dependence between the number of dumpsites and various social attributes from a spatial and temporal perspective. We perform large-scale global dumpsite detection and statistical analysis in areas out of the dataset. Spatially, we conduct a statistical analysis on the number of dumpsites on the satellite images of the central areas of 28 cities around 2021 and performed correlation analyses with 18 attributes obtained from official public data such as the World Bank and the United Nations. Several factors, such as different continents, level of development, population, and latitude, are considered to make the 28 cities globally representative. Every continent is included in our scope, and countries within the same continent are significantly different from each other in terms of development. Spatial analysis results reveal that the quantity of illegal dumpsites is relevant to development, urbanisation, sanitation, while interestingly, not statistically related to population, education, and technology. Temporally, we select central areas of several typical cities on multiple continents in our discussion, namely Munich, Tokyo, Kampala, and Shanghai, from 2015 to 2019 and tried to describe their changes. The time-series changes in those areas hint at the influence of policies like waste classification on the formation of illegal dumpsites. Overall, it is the first time researchers can detect classified dumpsites globally in an efficient and automated manner, as well as demonstrate a global correlation between the number of dumpsites and economic development, urbanisation progress, sanitation management, and policies.

In this work, our contributions are four-fold:

- We build a global fine-grained dumpsite detection dataset which can be used to train efficient deep learning networks.
- We design a novel deep convolutional model, BCA-Net, based on the characteristics of dumpsites. BCA-Net can detect more than 98% of dumpsites.
- We detect the number of dumpsites in the central areas of 28 cities based on our method and validate the generalisation of our method.
- We uncover statistical correlations between the number of dumpsites and development, urbanisation and sanitation.

## Results

The result is expanded into two parts: validation of our approach and spatial analysis in a global view with this approach.

### Validity and rationality

The illegal dumpsite’s composition is complicated, and its shapes are irregular, posing significant challenges to the deep model. In addition, the uneven distribution of the number of various categories in the training set will also reduce the overall performance. In order to prove that the proposed model can effectively detect dumpsites, we illustrate the model’s performance in this part. We split the dataset into a training set, a validation set and a test set in the typical ratio of 60:20:20^39–41^. Both *k*-fold (*k* = 5) and early stopping techniques are applied to increase the robustness of the results. Due to the problem of severe sample imbalance in the dumpsite dataset (Fig. 1a), we propose two training strategies, data augmentation (vertical flipping, horizontal flipping, forward 90° rotation and reverse 90° rotation) and category balancing, to ensure the model’s efficiency during the training process (see the methods and supplementary materials for details). Two issues in dumpsite detection are very worthy of attention. The first and most important thing is the sensitivity of the model to illegal dumpsites. The higher this sensitivity, the more illegal dumpsites can be detected. The second aspect of model evaluation is precision. The higher precision of the model for dumpsites means fewer misjudgments will appear in prediction results. Here, the sensitivity and precision of the model to the dumpsite are defined as:

1 $${{{{{\rm{Sensitivity}}}}}}=\frac{{{{{{\rm{No.}}}}}}\,{{{{{\rm{correctly}}}}}}\,{{{{{\rm{predicted}}}}}}\,{{{{{\rm{dumpsites}}}}}}}{{{{{{\rm{No.}}}}}}\,{{{{{\rm{all}}}}}}\,{{{{{\rm{labelled}}}}}}\,{{{{{\rm{dumpsites}}}}}}}$$

2 $${{{{{\rm{Precision}}}}}}=\frac{{{{{{\rm{No.}}}}}}\,{{{{{\rm{correct}}}}}}\,{{{{{\rm{dumpsite}}}}}}\,{{{{{\rm{predictions}}}}}}}{{{{{{\rm{No.}}}}}}\,{{{{{\rm{all}}}}}}\,{{{{{\rm{dumpsite}}}}}}\,{{{{{\rm{predictions}}}}}}}$$

Typically, higher sensitivity in geospatial detection requires a higher model tolerance, which means that the results are more likely to have lower precision. Table 1 illustrates the results of the model on the test set. Although global dumpsites’ irregular shape and appearance pose tremendous challenges to deep learning systems, our model still has an average sensitivity of over 98%. Interestingly, previous work^42^ demonstrates that humans have a sensitivity of 94.9% for image classification, yet our model achieves a sensitivity of 98% over that of humans in more challenging image detection (98% is based on 100% sensitivity of the annotators, which is demonstrated by the annotation process in supplementary materials). As the differences between distinct types of dumpsites are small and the same type of dumpsites can have significant morphological differences, the precision of the model has the potential to be improved with the advancement of satellite resolution in the future. It is worth noting that our model can be deployed to a personal laptop in a very short time and locate the dumpsites in the whole test set (162 square kilometres) in <30 s. Combining the sensitivity and precision of the model, we convert the high-cost method of manually positioning dumpsites into a low-cost and automated approach.

**Table 1:** Model performance on four types of dumpsites

	Sensitivity	Precision
Domestic waste	0.975	0.680
Construction waste	0.982	0.593
Covered waste	0.991	0.967
Agricultural waste	0.973	0.558
Average	0.980	0.701

**Fig. 2:**
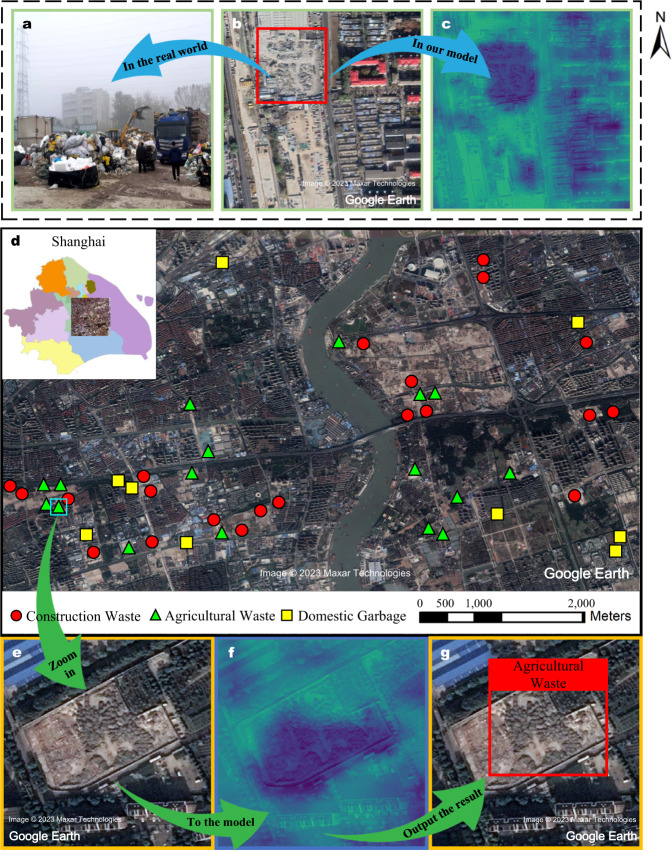
Presentation and confirmation of dumpsite detection results. **a**–**c** Photo of a domestic waste dump located in Beijing and its feature distribution in the model, which are shown with the owner’s permission. Supplementary Figure 6 provides an enlarged view of the highlighted areas in **c**. **d** Geographic distribution of dumpsites in the selected area in Shanghai. **e**–**g** Our model extracts the characteristics of an agricultural waste and uses a rectangular box to mark its location.

Figure 2 shows the authenticity and rationality of our method. In order to ensure the authenticity of our detection results, we confirm some dumpsites found in satellite images on the spot. Figure 2a, b are the real photo and the satellite image of the same domestic waste found in Beijing. Figure 2c, f show the “class activation map” (CAM)^43^ of the model in the inference process. The brighter part represents the possible location where the model predicts the dumpsite may exist. It can be seen from Fig. 2 that the trained model focuses on the interior and edges of the dumpsite. Figure 2b shows a car park (Supplementary Fig. 6 provides an enlarged picture), which looks similar to dumpsites. The model is also interested in this area, which has been shown with a light red area underneath the dumpsite in Fig. 2c. But due to the model’s sensitivity to detailed features, the area is ultimately not misclassified as a dumpsite because of the low confidence level. CAM reveals the internal working mechanism of the deep learning model and also explains the rationality of this approach, which is also detailed in supplementary materials (see “Insights through the BCA-Net” section). Figures 2d–g illustrate the flow of our method and the detection results at the regional level. Each dumpsite in subsequent results goes through the process of Fig. 2e–g to obtain the final location box and category label.

### Spatial analysis

**Fig. 3:**
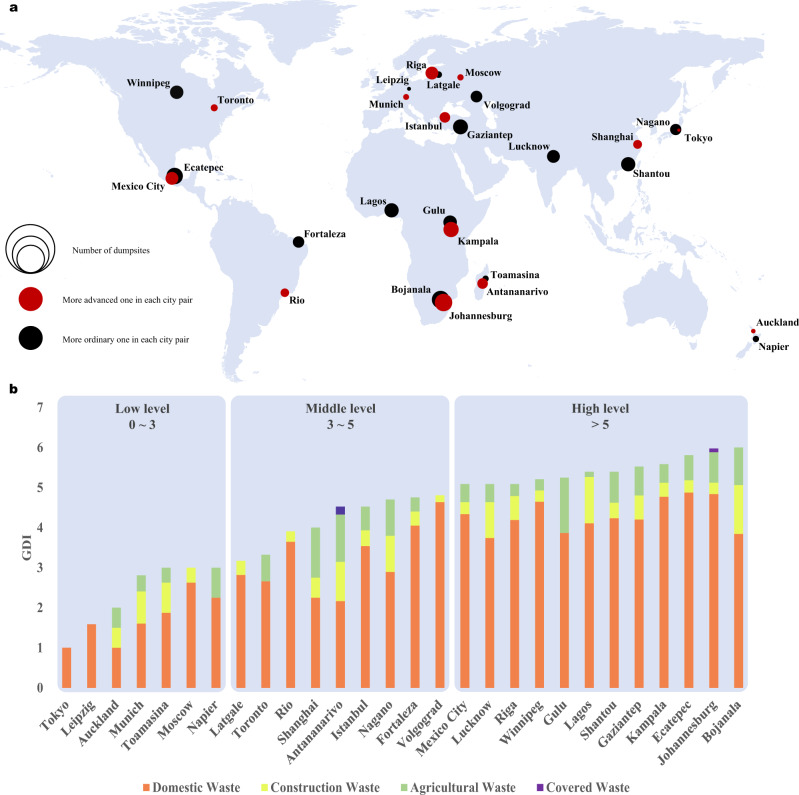
Geographical distribution and Global Dumpsite Index (GDI) of selected areas for spatial statistics experiments. **a** Global distribution and quantity comparison of 28 areas for spatial analysis. Red and black circles represent two areas of the same country, and the area of the circle represents the number of dumpsites. **b** GDI and percentage of different types of dumpsites in 28 areas. The order is determined by GDI, and different colours represent different types of dumpsites.

To analyse the relationship between illegal dumpsites and social attributes, we conduct extensive dumpsite detection and statistics with a global view to explore the potential conditions that affect the formation of dumpsites. Considering the latitude, development level, health ranking, income level, etc., we select 28 areas out of the dataset with a large population of 15 countries for quantitative analysis. In addition, we investigate two areas in most countries to analyse the difference between areas with different levels of development within a country. One of them is a well-developed metropolis, and the other is an ordinary city with a large population (only one city is investigated in India and Nigeria considering the accessibility of suitable remote sensing images). Analysis materials are satellite images of these areas around 2021 (about 160 square kilometres per area). Supplementary Figures 7–13 mark all dumpsite locations in several areas. Figure 3a presents the geographical distribution of 28 areas. Red and black represent the relatively progressive and ordinary city in each city pair, and the circular area reflects the number of dumpsites detected in an area. In order to construct a simple and intuitive global evaluation indicator, we propose a new Global Dumpsite Index (GDI) to represent the quantitative level of dumpsites in city centre areas. GDI is positively correlated with the number of dumpsites (logarithm of the number of dumpsites to the base 2), and the details of GDI will be discussed in the method part. Figure 3b shows the GDI in 28 areas and the percentage of different dumpsites (Supplementary Fig. 9 shows specific numbers). Figure 3a illustrates that the total number of dumpsites in more progressive cities (within the same country) is relatively small and that in more developed countries is relatively small as well. These two phenomena suggest that the more advanced the area, the smaller the probability of the formation of dumpsites.

To explore which factors are related to the number of illegal dumpsites, we analyse the correlation between the number of dumpsites and 18 valid attributes of the selected cities or countries obtained from official public data such as the World Bank and the United Nations. Supplementary Table 10 shows the values and sources of the 18 attributes. Table 2 shows the name of 18 social attributes and their Spearman correlations with the number of dumpsites. Spearman correlation has been widely used in correlation analysis^44–48^. In detail, the analysis results of total dumpsites (ALL), domestic waste (DW), construction waste (CW) and agricultural waste (AW) are provided. The mark * in Table 2 indicates a statistical correlation between two variables, while ** indicates a strong correlation (see Supplementary Table 11 for more details). The results demonstrate that the total number of dumpsites is statistically correlated with the urbanisation process, income level, developed or developing country, latitude, and health ranking. The number of single-type dumpsites is mostly linearly related to these attributes as well. Statistical results can be listed as follows:

- Development: countries with higher development levels may have more mature dumpsite treatment plans, which can help avoid the formation of large dumpsites and make cities tidier.
- Urbanisation: process of urbanisation affects the formation of dumpsites to a certain extent. Normally, more urbanised countries have stricter regulations on city appearance and are less likely to form a massive dumpsites. Conversely, less urbanised areas with fewer regulations may have a higher tolerance for large dumpsites.
- Sanitation: the number of dumpsites shows a strong statistical correlation with the national sanitation level ranking, which implies that the number of dumpsites can also be an indicator to measure the health level of a region/country.
- Others: interestingly, there is no direct correlation between the number of dumpsites (in city centres) and population, education rankings, and technology rankings. This finding suggests that population, education, and technology level do not ultimately impact the number of dumpsites in city centres. We speculate that the government’s policy regulation and implementation may have a more profound impact on the number of dumpsites.

**Table 2:** The spearman correlation analysis results of 18 attributes and the number of dumpsites

	All	DW	CW	AW
Urbanisation ranking	0.418*	0.351	0.37	0.381*
International innovation index	0.311	0.294	0.222	0.124
Gross national income	0.538**	0.480**	0.486**	0.455*
Developed country or not	−0.554**	−0.536**	−0.403*	0.420*
Waste treatment recycling (%)	0.133	0.109	0.117	0.218
Waste treatment incineration (%)	−0.488	−0.515	0.103	−0.323
Waste treatment open dump (%)	−0.214	−0.273	−0.044	−0.161
Municipal solid waste	0.036	0.071	0.037	−0.053
Global waste index	−0.381	−0.395	−0.254	−0.298
Absolute latitude	−0.424*	−0.365	−0.312	−0.463*
Latitude	−0.204	−0.144	−0.149	−0.377*
Northern/Southern hemisphere	−0.083	−0.118	0.025	0.074
GDP per capita	−0.356	−0.353	−0.230	−0.455*
National GDP	0.096	0.049	0.269	0.187
Population	−0.099	−0.073	−0.129	0.032
Population density	−0.188	−0.188	0.090	−0.108
Cleanest countries in the world	0.487**	0.424*	0.475*	0.433*
Education rankings	0.366	0.351	0.382	0.294

The values in the table are correlation coefficients (not *P*-values). Only variables marked with * or ** in the table are statistically relevant, while other correlations are not statistically relevant. *P* < 0.05 indicates a statistically linear correlation, which is marked with *. *P* < 0.01 indicates a strong correlation, which is marked with ** (Supplementary Table 10 includes the *P*-values of these correlations).

The spatial analysis demonstrates that our method can be a quantitative tool for evaluating regional dumpsite management. By comparing the absolute number and distribution density of dumpsites in multiple regions, the government can analyse the dumpsite management in these regions, compare them from a policy perspective and even give guidance. We believe that this automated method will greatly optimise the management of dumpsites. Furthermore, an intelligent management system will significantly reduce carbon emissions and infectious diseases around dumpsites as well as improve their recycling rate and sustainability.

## Discussion

This section will discuss the factors that influence the formation of dumpsites from a temporal perspective. In addition, we also summarise the contribution and potential significance of this work.

### Temporal discussion

**Fig. 4:**
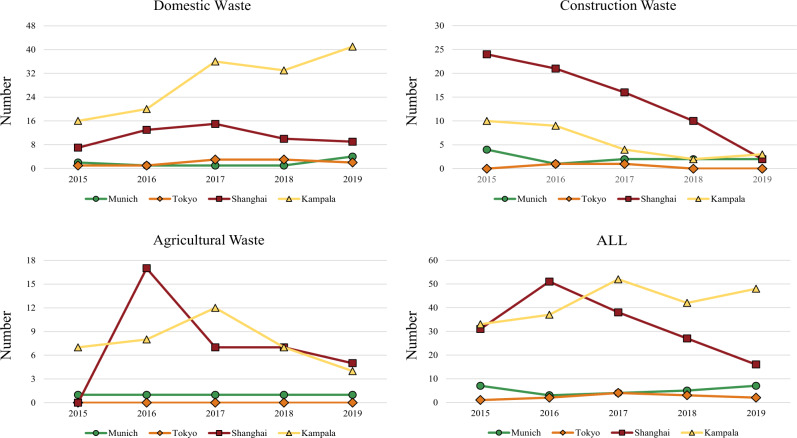
Changes in the three types of dumpsites and the total number of dumpsites in the four selected cities from 2015 to 2019. The four tables record the trends of Domestic Waste, Construction Waste, Agricultural Waste, and total dumpsites in these areas from 2015 to 2019.

Here, we attempt to discuss which human factors may have relationships with the formation of illegal dumpsites from a temporal perspective. Specifically, four representative areas are selected as discussed areas: Munich in Germany (48° 7′55.46″N 11° 34′52.01″E, about 156 square kilometres), Tokyo in Japan (35^∘^42′12.07″N 139^∘^42′ 47.65″E, about 158 square kilometres), Shanghai in China (31^∘^9′5.90″N 121^∘^27′19.98″E, about 166 square kilometres), Kampala in Uganda (0^∘^19′30.66″N 32^∘^35′23.61″E). Each of them is one of the most advanced cities in the country, but they have significantly different waste management policies. Munich and Tokyo have well-developed waste management policies, and Shanghai has recently been optimising its management policies, while Kampala’s waste management policies are relatively poor. Given the possible influence of resolution on the results (see supplementary materials), we set a uniform resolution of 0.6 m for the satellite images used in this section. Figure 4 shows the trend of three types of dumpsites and the total amount of dumpsites in five years. The results of Fig. 4 are summarised as follows with our discussion:

- There are relatively more dumpsites in Shanghai and Kampala. Over time, the number of dumpsites in Shanghai is slowly approaching Tokyo and Munich, while Kampala seems to be maintaining an overall upward trend. Relevant materials mention that Shanghai implemented a large-scale urban renewal programme in 2014^49^, and many old buildings have undergone demolition and reconstruction since that year. From Fig. 4, many “construction waste” and “agricultural waste” existed from 2015 to 2016. In addition, Shanghai began to advocate a waste classification plan in 2018, which was officially implemented in 2019. The amount of “domestic waste” in this area had maintained an upward trend from 2015 to 2017 while decreased significantly from 2017 to 2019, which may reflect a possible link between the waste classification policy and the number of dumpsites to a certain extent. For Kampala, scholars stated that poverty and backward consciousness have greatly restricted Kampala’s waste management revolution around 2020^50,51^. Most residents do not segregate their waste, which leads to a low waste collection rate. Moreover, the inefficient implementation of waste management policies also caused the formation of dumpsites^50^. The joint placement of medical waste and other waste poses a certain safety hazard to surrounding animals and humans^51^. The low urbanisation rate^50^ makes construction waste less, but domestic waste in Kampala exceeds the sum of the other three cities and continues to rise. According to the above literature, the relative backwardness of the Kampala government’s waste management policies and supervision may be one of the social factors behind these big numbers. Based on our findings in the spatial analysis, poor urbanisation and economic development factors also contribute to the formation of dumps in Kampala. It is not easy to analyse the many social factors of dumpsite formation, and more supportive data may be available to study them in the future.
- Munich and Tokyo, which are also large and advanced cities like Shanghai, have far fewer dumpsites than Shanghai, reflecting from a macro perspective that these two selected areas’ environmental governance and urbanisation are better than Shanghai based on our previous conclusions. According to the census, the population density of Tokyo, Japan, and Munich, Germany in 2020 were about 6363 and 520 people per square kilometre, respectively. However, the number of dumpsites in the selected area of Tokyo is less than that of Munich, suggesting that the selected area of Tokyo does well in urban dumpsite management to some extent. From the perspective of waste management policies, the Japanese and German governments entered the era of waste sorting by formulating environmental-related legal documents earlier than most countries^52,53^ and have achieved satisfactory results in treatment and recycling of waste even in 2000, which might be a potential promoting factor to the stationary low dumpsite distributions in the inspected period from 2015 to 2019. It will be interesting to study the correlation between effective waste treatment policies (especially waste classification and circular economy) and the number of dumpsites, which is one of the studies we are planning.

**Fig. 5:**
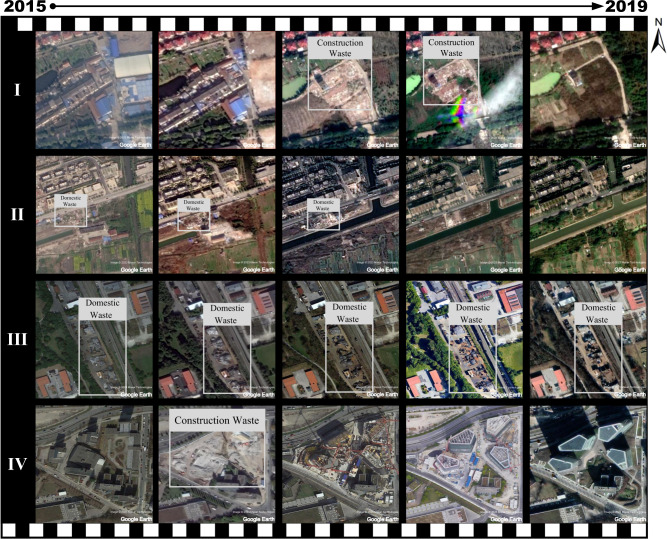
Changes in the location of four dumpsites in five years. Line I, Demolition of houses forms construction waste, which is then built into lawns. Line II, Illegal dumpsites are cleared and built into a green belt. Line III, A dedicated dumpsite. Line IV, Construction waste formed by the demolition of old houses were subsequently built into office buildings.

After observing a large number of dumpsites, we are very interested in the reasons for the formation of dumpsites and their future changes, so we display the five-year changes in the location of four dumpsites in Fig. 5. Line I illustrates that a piece of construction waste in Shanghai was formed as the house had been demolished and disappeared as the lawn had been built. Line II shows an illegal dumpsite in Shanghai that had been transformed into a green belt in 2019. The formation of dumpsites in Line I reflects the implementation of the large-scale urban renewal programme, while the two dumpsites in Line I and Line II finally turned into green spaces, which is in line with Shanghai’s green space regulations. Line III shows a dedicated dumpsite in Munich that has not changed much in five years. Line IV displays an old building demolished and replaced by a modern office building in Munich. The time-series discussion suggests that large-scale urban construction programmes and the implementation of waste classification policies may potentially affect the number of dumpsites.

### Other discussions

We summarise the main contributions of this work and discuss some difficult details here. Firstly, we hope to elaborate on the difficult details of this work. Results in Table 1 suggested that the precision still has room for improvement in the future, which makes the low-cost manual screening phase take more time (even though the time is much less than previous methods). In fact, two factors limit this improvement. The first is the resolution limit of current satellite imagery. The resolution of the quality satellite images that can be obtained so far is mostly from 0.3m to 1m per pixel, which means that only the macro characteristics and approximate edge information of the dumpsite can be obtained from the satellite images, but the internal details cannot be distinguished clearly. This limitation makes the model treat a small number of objects similar to the macroscopic characteristics of dumpsites as dumpsites (such as the roofs of high-rise buildings, reflections on the sea surface, etc.). The second factor is that due to the influence of various cultures, dumpsites in different countries do not have a consistent appearance, which makes the contents of dumpsites more complicated with irregular shapes (Please see the Model part in the Methods section for details). Both of the above factors lead to the low precision of the model. The low precision does not affect the model’s sensitivity to dumpsites, only that the results require a small amount of manual sifting, which is still very efficient compared to relying entirely on humans.

Nevertheless, our approach can detect nearly all dumpsites and save most of the time compared with the manual census. For example, we spent six months searching and classifying dumpsites on satellite images (about 4800 square kilometres), while the model only takes less than an hour to complete the process of searching and classifying in central areas of nearly 30 cities (about 5000 square kilometres of satellite images). Including the time spent on manual rechecking of the model results, our approach takes about six days to search and classify dumpsites on these satellite images. In other words, this approach saves 96.8% of the time compared to manual labelling. Moreover, the supervision of dumpsites is a global work, which means every country needs to regularly locate and count dumpsites. It may take several years to scan the satellite map of a single country manually^54^, so global scanning is almost impossible to achieve manually. However, an automated approach can capture 98% of the dumpsites and classify them correctly in a very short time, which provides a powerful solution to obtain the location of dumpsites worldwide.

To increase the confidence of our method, we further investigate the authenticity of many dumpsites in our dataset through field visits and inquiries. We present many photographs taken during the fieldwork and their corresponding satellite images in Fig. 2a and Supplementary Figs. 4 and 5. In addition, Supplementary Table 8 shows our extensive field visits in China between December 2020 and May 2021. For dumpsites out of China, we search help from familiar scholars in Germany and Japan to identify. Furthermore, we use VR tools like Google Street View to verify hard-to-reach dumpsites as much as possible due to the COVID-19.

In addition, our approach can also be improved to analyse some other interesting things. As shown in Fig. 5 Line I and IV, the construction waste area generally had construction activities in those years. The land changes before and after construction waste can reflect the land use planning of those areas in recent years. Similar methods can also be used to study issues like urban construction efficiency, urban construction intensity and even the urban greening process.

With the further improvement of satellite imaging techniques and deeper implementation of waste classification policy, it will be possible that dumpsite detection and classification can be fully automated, and we will follow up.

## Methods

### Data

According to the world city population rankings in the 2016 World Cities Report^34^ and the Global National Environmental Performance Index (EPI)^35^ rankings released by Yale University in 2020, we selected several typical cities to build the dataset. These selected cities have relatively larger populations and relatively poor environmental performance, which is conducive to forming a large dataset. They are Colombo in Sri Lanka (EPI 109/180), Dhaka in Bangladesh (EPI 162/180), Guwahati in India (EPI 168/180), Kinshasa in the Democratic Republic of Congo (EPI 125/180), Lagos in Nigeria, New Delhi in India, and several cities in China (EPI 120/180).

We spent almost six months from December 2020 to May 2021 with twelve experts on these satellite images to thoroughly search and label the dumpsites. The PASCAL VOC data annotation format^55^, which is often used in computer vision, is selected as the annotation format of the dumpsite dataset. We performed a series of preprocessing on images of the dataset. Considering the memory consumption of the model and some other factors, we divided the large-area satellite images into 2219 tile images size of 1024 × 1024 pixels, and the area of each picture is about 614 × 614 m^2^, which is enough to hold a giant dumpsite. For the same city, the image resolution is the same. After that, we classified all dumpsites without exception into domestic waste, construction waste, agricultural waste, and covered waste, depending on the different sources and forms of distribution. Typically, domestic waste is small and complex, while construction waste, agricultural waste, and covered waste have relatively distinct characteristics. (All images and the annotations in this work can be used for academic purposes only, but any commercial use is prohibited.)

### Model

For the model, we propose a new deep convolutional network, Blocked Channel Attention Network (BCA-Net). In order to effectively extract features from the dumpsite dataset, the two-stage^56^ object detection network, Faster-RCNN, which has been widely used in computer vision^57–60^, is employed as a baseline model for learning dumpsites. Faster-RCNN extracts features from the training set through the multi-level convolutional encoder of the backbone network and then delivers the features to the Region Proposal Network (RPN). After that, regression and SoftMax loss are designed in the RPN network to learn the parameters of the background classifier and the foreground bounding box regressor. Finally, the features extracted by the backbone network, combined with the foreground classification detection results of the RPN, are sent to the final object classification and bounding box regression module. Similarly, two losses are designed to obtain more refined bounding boxes and classification results.

The size of the dumpsite in our dataset varies from hundreds to thousands of square metres, which means the area of a large dumpsite may be several hundred times that of a small one. However, Faster-RCNN only uses the high-level features of the last several stages so that features of smaller dumpsites are often missed after multiple convolution operations, which makes this network structure insufficient to detect small dumpsites. To avoid this weakness, the Feature Pyramid Networks (FPN) structure^61^, which aims at preserving multi-scale features of dumpsites, is added to our model. Supplementary Table 6 demonstrates that although the model has added multiple layers of feature information, the training process can still be completed within a few hours.

When observing the inference results, we found that existing models could not solve two difficulties in the dumpsite dataset. On the one hand, the colours of dumpsites in satellite images are often similar to their background environment, and the boundaries of dumpsites differ significantly from their inner parts, which make it challenging to define the edge of the dumpsite and the background environment in the satellite images. On the other hand, the morphological characteristics of several types of dumpsites are not significantly different in the macroscopic view as shown in Fig. 2, which means it is difficult to distinguish the correct category without observing the detailed features from multiple aspects. In deep convolutional models, each output channel in each layer computed during the feature extraction process represents the model’s understanding of the dataset in different perspectives. Even different regions within the same channel have features with different importance. However, conventional models, including the Faster-RCNN-FPN, SE-net^62^ and CBAM^63^, do not consider the different importance of these features. In other words, the importance of all N regions in different channels are the same, which means the proportion coefficients are all 1/N. Consequently, even if the model could extract important characteristic information of dumpsites in specific regions of some feature channels, such typical information will also be concealed by comparatively indistinguishable features during the inferring process. This limitation will reduce the performance of the model on the dumpsite dataset.

Therefore, the “Blocked Channel Attention” (BCA) module is introduced to emphasise the critical feature information in feature channels. The concept of attention first appeared in the field of Natural Language Processing^64^. In recent years, attention-based methods have also been used in computer vision, including SE-Net, CBAM, etc., which positively affect the conventional structure. SE-Net is one of the pioneers of attentional mechanisms in computer vision, whose attention layers exist on different convolutional channels. SE-Net has a small size and is plug-and-play for most convolutional structures. CBAM adds attention mechanisms to both the channel and spatial dimensions. For channel attention, CBAM uses a similar structure in SE-Net. For spatial attention, CBAM turns each *C* × *H* × *W* layer into two 1 × *H* × *W* features through a pooling operation. After that, spatial attention is computed by convolution operations. However, both remarkable attention mechanisms fail to distinguish the importance of different features within the same channel mentioned above. Thus, we try to improve the understanding of irregular dumpsites by simultaneously computing channel and spatial attention of the proposed model rather than computing in two steps.

**Fig. 6:**
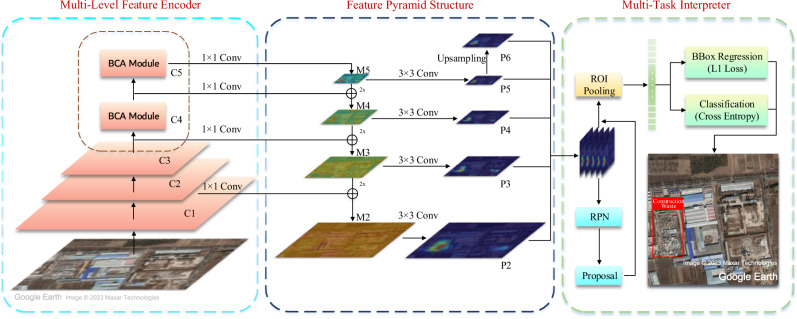
The detailed structure diagram of BCA-Net. Specifically, BCA-Net adds “Blocked Channel Attention” modules to the high-level feature layers of ResNet50 (C4 and C5 layers).

**Fig. 7:**
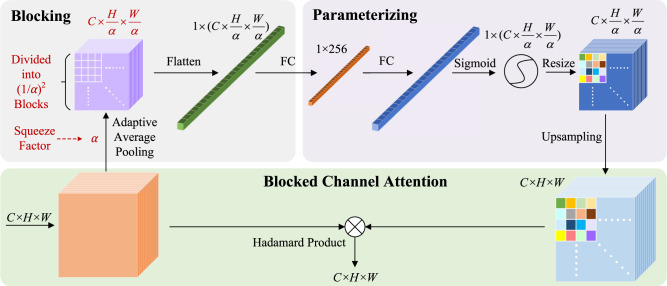
The specific structure of the BCA module. The blocking part vectorises three-dimensional features, and the parameterising part gives BCA the ability to learn. The last part distinguishes the importance of three-dimensional features according to different regions.

When designing the BCA module, the space occupied by the model parameters and the actual improvements are considered. Thus, only the last two residual feature modules of the feature extraction network ResNet50^65^ are replaced with the BCA module, as shown in Fig. 6. Specifically, the BCA module divides all *H* × *W* channels in the selected residual modules into several blocks, and the hyperparameter squeeze factor *α* determines the number of blocks. As shown in Fig. 7, the number of blocks obtained after segmentation of each channel feature map is:

$$\frac{H\times W}{{\alpha }^{2}}$$

Thus, the number of all blocks is:

$$\frac{C\times H\times W}{{\alpha }^{2}}$$

After blocking, all blocks are flattened into a one-dimensional vector to obtain the importance weights of different blocks. Then the 1-D vector is linked to a fully connected layer containing 256 neural units to obtain more representative high-level information. After that, the 256-D FC layer is linked to an original sized fully connected layer to restore the size, as well as extract the importance distribution information in the 256-dimensional high-level parameter space. Since the blocking and flattening operations cannot introduce trainable parameters while the fully connected layers can, these two FC layers also parameterise the BCA modules, enabling them to learn like other parts of the model in the iterative process. The last two steps of “parameterisation” are the commonly used non-linear operation “sigmoid” and the resize operation that transforms a one-dimensional vector into a matrix. The purpose of these two steps is to improve the model function’s representability and map the attention weights to the original *C* × *H* × *W* feature correspondingly. Finally, obtain the Hadamard product of the attention weights matrix and the original feature residual modules matrix to complete the BCA module calculation. In order to illustrate the improvement brought by BCA-Net, we conduct several sets of comparison and ablation experiments in supplementary materials. Network models involved in the comparison include our previous structure SRAF-Net^66^, Faster-RCNN-FPN, SE-Net^62^, CBAM^63^ and BCA-Net. Supplementary Figures 1–3 and Supplementary Tables 1–7 illustrate the results of ablation experiments with data and examples. We set the Non Maximum Suppression (NMS) threshold to 0.001 to detect as many objects as possible in the spatial analysis and temporal discussion.

### Training details

Data augmentation and category balancing strategies are applied to the training process to avoid the effects of long-tailed distributions^67^ on the results (see supplementary materials for the concept and practical effects of long-tailed distributions). Specifically, the data augmentation methods include vertical flipping, horizontal flipping, forward 90° rotation and reverse 90° rotation. The category balancing strategy maintains the same probability of each class appearing in a batch during training.

The *k*-fold technique (*k* = 5) is used to perform multiple rounds of validation. For hyperparameters, the learning rate is set as 0.0025. Stochastic Gradient Descent (SGD) is selected as the optimiser with a momentum of 0.9 and weight decay of 0.0001. The learning rate is decayed at epochs 16 and 22. We set the maximum epoch to 24 and implement the early stopping technique based on the performance of the validation set. A linear warm-up technique is also implemented for the first 500 iterations with a warm-up ratio of 0.001 (see supplementary materials for details). All experiments are carried out on eight NVIDIA RTX3090 graphics processors.

### Dataset universality

In this work, we first search for dumpsites in seven countries and built a dumpsite dataset. Extensive experiments in supplementary methods demonstrate the strong sensitivity of our model to dumpsites. We then leverage the model to detect dumpsites in 28 urban areas worldwide. To verify that the dataset has sound detection capability for global dumpsites, we manually search satellite images of these 28 regions without using any additional methods and obtain 763 dumpsites. Meanwhile, we obtain a total of 755 dumpsites (98.6%) with the methodology of this work. Such results show that our dataset has a good global generalisation. We also investigate the appearance and origin of dumpsites around the world through media, internet, and mail enquiries. Due to the minor differences between dumpsite formations, most forms of global dumps in satellite imagery are included in our dataset.

### Details of GDI

We design an index to intuitively reflect the level of dumpsites in a region with a global view, and we define the GDI as the logarithm of the number of dumpsites to the base two (see supplementary materials for design intent). The calculation formula of GDI is as follows:

3 $$GDI={\log }_{2}\,N,$$

and *N* denotes the number of dumpsites. Then, we sort all regions into three levels from low to high, and their GDI ranges from 0 to 3 (low level), 3 to 5 (middle level), and >5 (high level). It can be inferred from Fig. 3b that cities with better development levels generally have lower GDIs. Theoretically, GDI can represent the level of dumpsites in a region on a global scale.

## Supplementary information

Supplementary Information

## Data Availability

The replication dataset generated during the current study is available in Science Data Bank.

## Code availability

The code used to train and evaluate the model is available on GitHub^68^.
