# Supplementary material for: Revealing influencing factors on global waste distribution via deep-learning based dumpsite detection from satellite imagery
Source: Nat Commun. 2023 Mar 15;14:1444. doi: 10.1038/s41467-023-37136-1 (PMC10015540; doi:10.1038/s41467-023-37136-1)
Supplement: Supplementary file 1 — Supplementary Information [file 41467_2023_37136_MOESM1_ESM.pdf]

Revealing influencing factors on global waste distribution via deep-learning based dumpsite detection from satellite imagery

Xian Sun, Dongshuo Yin et al.

Supplementary Methods

Model optimization: BCA-Net

In order to effectively extract features from our dumpsite dataset, we propose BCA-Net to detect the dumpsite for better performance. Before introducing the new module, we will briefly review the baseline structures. The Faster R-CNN [1] object detection network is used as a baseline model for learning dumpsites. Faster R-CNN extracts features from the training set through the multi-level convolutional encoder of the backbone network and then delivers the features to the RPN network. The ResNet [2] feature extractor, one of the most recognisable convolutional backbones, is often used in the Faster R-CNN detection framework. Our BCA module is also optimised based on ResNet. Specifically, we use ResNet50 as the backbone for all models. After RPN, regression and SoftMax loss in the RPN network can help train the parameters of the background classifier and the foreground bounding box regressor. Finally, the features extracted by the backbone network, combined with the foreground classification detection results of the RPN network, are sent to the final object classification and bounding box regression module.

Dumpsites in our dataset usually vary in size. For example, a small "domestic waste" may be dozens or even hundreds of times smaller than a giant "construction waste". However, Faster R-CNN only uses the high-level features of the last several stages of the encoder, leading to the loss of smaller dumpsite after multiple convolution operations. Tsung-Yi Lin et al. [3] propose the Feature Pyramid Networks(FPN), which sends all levels of convolutional features in the encoder as parallel-styled features to subsequent modules and obtain their respective prediction results. Such a hierarchical structure undoubtedly increases the time of training and inference, but the information of the small dumpsite is better preserved due to low-level features. Certainly, our BCA-Net is also inspired by FPN.

There are two phenomena in our dumpsite dataset. Firstly, the dumpsites in satellite images are often blurred with their background, and the boundaries of the dumpsites are usually quite different from the inner appearance. This fact makes it hard to distinguish the boundary of the dumpsite and the background in satellite images. Secondly, as shown in Figure 1 (d) (body part), the dumpsites of different types have similar morphological characteristics in the macroscopic view. It is difficult to distinguish the correct category without observing the detailed features from multiple aspects. In Deep Convolutional Neural Network (DCNN) models, each channel in each layer computed during the feature extraction process represents the model's understanding of the dataset from different perspectives. Even different regions within the same channel have different degrees of importance in feature understanding. However, conventional models, including the ResNet50 here, do not consider the different importance of these features. In other words, the importance of all N regions in different channels is the same, and the proportion coefficients are all 1/N. Consequently, even if the model could extract important characteristic information of the dumpsite in specific regions of some feature channels, such typical information will also be concealed by comparatively indistinguishable features during the inferring process. This limitation will reduce the performance of models on the dumpsite detection.

Considering the above two phenomena, we propose the "Blocked Channel Attention" (BCA) module to emphasize the critical features in all feature channels. The concept of attention first appeared in the field of Natural Language Processing [4]. In recent years, attention-based methods have also been used in computer vision, including SE-Net [5], CBAM [6], etc., which positively affect the conventional structure. SE-Net is one of the pioneers of attentional mechanisms in computer vision, whose attention layers exist on different convolutional channels. SE-Net has a small size and is plug-and-play for most convolutional structures. CBAM adds attention mechanisms to both the channel and spatial dimensions. For channel attention, CBAM uses a similar structure to SE-Net. For spatial attention, CBAM turns each $C \times H \times W$ layer into two $1 \times H \times W$ features through a pooling operation, after which attention is computed by convolution. Interestingly, CBAM's channel and spatial attention are computed in two divided steps. Inspired by CBAM, we began to think: would the model's understanding of irregular dumpsites be improved by simultaneously computing channel and spatial attention? We accordingly design the BCA module to try it out.

When designing BCA, the memory consumption of parameters and the actual improvement are taken into account. If BCA modules were added to all layers in ResNet50, the global attention calculation would consume a lot of resources, which would be difficult to perform on a personal computer. Thus, only the last two residual feature modules of ResNet50 are replaced with the BCA module, as shown in Figure 6 (body part). Specifically, the BCA module divides all $H \times W - sized$ channels of the selected residual feature modules into several blocks, and the hyperparameter squeeze factor α determines the number of blocks. As shown in Figure 7 (body part), the number of blocks obtained after segmentation of each channel feature map is:

$$\frac{H \times W}{\alpha^2}$$

Undoubtedly, all channels are divided into:

$$\frac{C \times H \times W}{\alpha^2}$$

blocks. Afterwards, all blocks are flattened into a one-dimensional vector to obtain the importance weights of different blocks. Then the 1-D vector is linked to a fully connected (FC) layer containing 256 neural units to obtain more representative high-level information. The 256-D FC layer is connected to an original sized FC layer to restore the size, and at the same time extract the importance distribution information in the 256-dimensional high-level parameter space. The blocking and flattening operations do not have trainable parameters while the FC layers have. So FC layers also parameterize the BCA module which makes BCA module can be optimaized like other parts in the iterative training process. The last two steps of "parameterization" are the commonly used non-linear operation "sigmoid" and the resize operation which transforms a one-dimensional vector into a matrix. The purposes of these two steps are to improve the representability of the model function and mapping the attention weights to the original $C \times H \times W$ feature correspondingly. Finally, obtaining the Hadamard product of the attention weights matrix and the original feature residual modules matrix to complete the BCA module calculation.

Long-tailed distribution problem

Figure 1 (a) (body part) shows the proportions of four types of dumpsites. "Covered waste" is only 1% of our dataset, while other types can be several or even tens of times more numerous. Given the importance of high-density polyethene (HDPE) films for permeability and odour management [7, 8, 9], we retain the category of "covered waste", even though there are less than 30 instances. Indeed, such extreme imbalance in categories is common in object detection. In the most recent large remote sensing dataset, MultiScene [10] has 36 categories, while its min/max number of instances of a single category is 22/8628. Moreover, SEN12MS [11] with 16 categories reaches 14/31836. These rare categories often play the same important roles as other categories. Also, unbalanced datasets require robust and comprehensive models and training methods.

During the experiment, it is found that the highly uneven distribution of the sample size makes the model unable to capture the characteristics of "covered waste". Specifically, the model performs very poorly, and the Average Precision (AP) of this category is almost zero (Supplementary Table 2). In the computer vision field, the "long-tailed" problem is commonly solved from two perspectives: resampling and reweighting [12]. The core idea of resampling is to increase the number of "tailed" samples by increasing the number of times that the model learns "tailed" data. Reweighting is to increase the weight of the "tailed" data in the loss function. In other words, the weight is used to increase the gradient of the "tailed" data in the backpropagation process. In order to break this deadlock in our dumpsite dataset, we also try to conceptualise the solution in two ways: (1) Resampling: Two methods based on resampling were proposed, namely: a) Apply data augmentation training strategy to extremely unbalanced "covered waste" to enlarge its number through vertical flipping, horizontal flipping, forward 90°rotation and reverse 90°rotation. b) In the training process, the training strategy of balancing sampling is used for the data of each batch. Specifically, each batch is generated in a balanced proportion according to the number of samples in each category. Here, the "covered waste" category is sampled in a balanced manner so that the model can learn its characteristics better. (2) Reweighting: Since the dumpsite dataset has only four categories, and the "covered waste" with the most uneven distribution has less than 30 samples, the use of typical reweighting strategy (Focal Loss) will significantly affect the normal parameter space, leading the space to be distorted and reducing the accuracy of all categories in dumpsite dataset. Experiments demonstrate that existing weighted loss function in computer vision is not suitable for the "cliff-typed" data distribution form of the dumpsite dataset (Supplementary Table 5).

Evaluation metric for model comparision

In order to evaluate the ability of the model to detect and identify dumpsites, the mean Average Precision (mAP) [13, 14] series, the most authoritative evaluation metric in the computer vision field, is used as the evaluation metric in this study. The mAP can simultaneously reflect the model's recall rate and precision rate according to its calculation process. In other words, the larger the mAP ($0 \sim 100\%$), the more dumpsites can be successfully detected and the lower the false alarms in all types of dumpsites. Here, the most widely used mAP50 in the mAP series is selected as the overall evaluation metric, and AP50 for each category. The calculation of the mAP series is a relatively complex and mature technique, and mAP is obtained by averaging APs of each category. Besides, AP_{Medium} and AP_{Large} are also included to observe the model's ability to detect different sizes of dumpsites. AP_{Small}, AP_{Medium}, and AP_{Large} are objects with sizes less than 32^2 pixels, $32^2 \sim 96^2$ pixels, and more than 96^2, respectively. AP_{Small} is not shown here as our dataset has no dumpsites smaller than 32^2 pixels.

Method comparision

This part is mainly advanced to demonstrate the effectiveness of the dumpsite dataset and BCA-Net by comparing the performance with other models. We conduct experimental demonstrations from three aspects: (1) Compare the performance of BCA-Net with Faster-RCNN-FPN and previous work on unclassified dumpsite dataset to explore the promotion of BCA-Net on the unclassified dumpsites detection task. (2) Conduct experiments on labeled dumpsite dataset to explore the feasibility of dumpsite classification and the performance of BCA-Net on this task. (3) Try to analyze and evaluate the model's ability to extract dumpsite features through visualizing the model's intermediate-layer features.

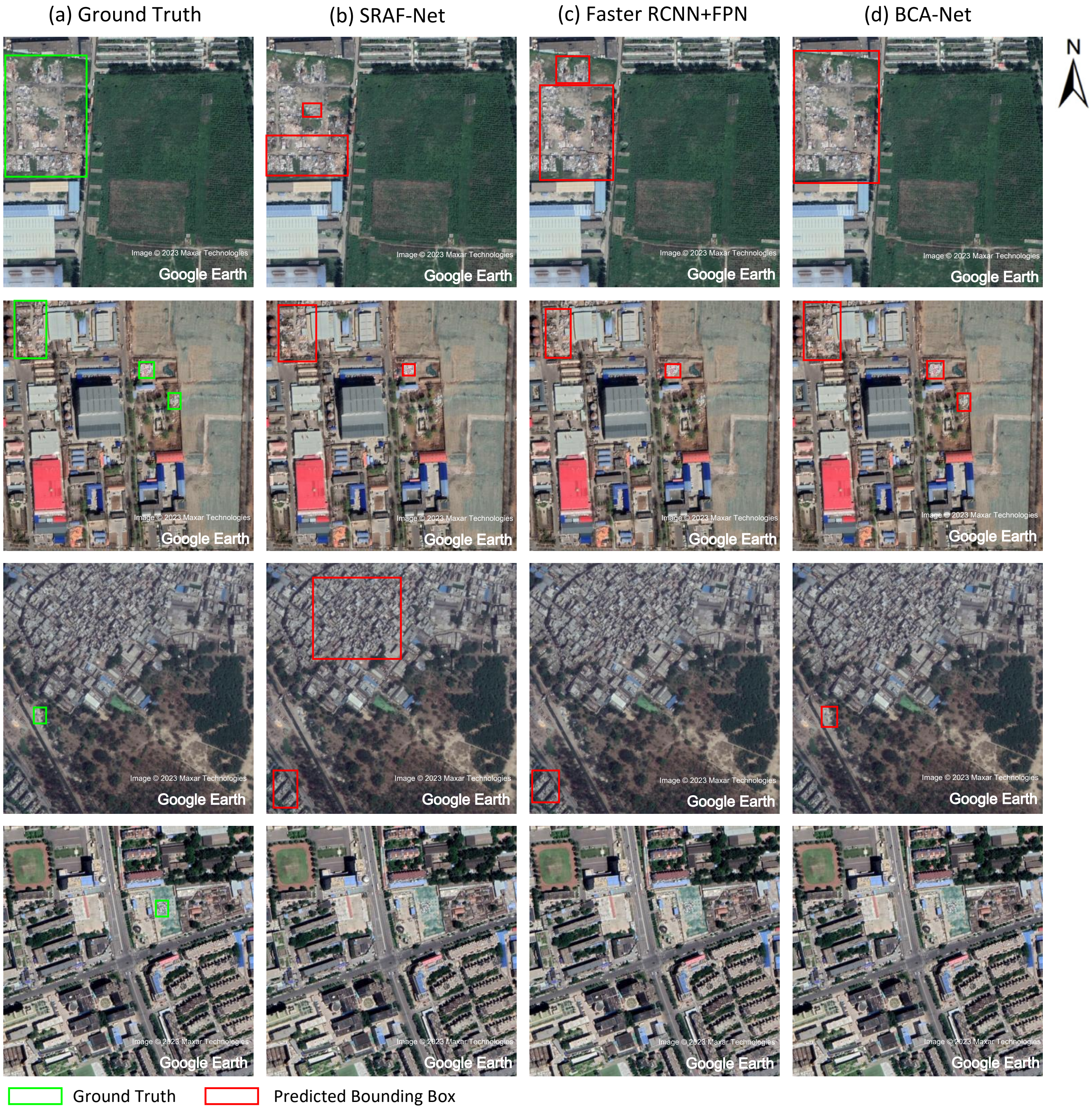

Supplementary Figure 1: (a) Ground truth of four examples. (b) Predicted results of SRAF-Net. (c) Predicted results of Faster-RCNN-FPN. (d) Predicted results of BCA-Net. All these images are from the test set.

For the dataset, we split it into a training set, a validation set and a test set according to 60:20:20. For the categorised dataset, we also separate the objects for each class in this ratio. The k-fold technique (k=5) is used to perform multiple rounds of validation, and then the average of the results is calculated as the final result. For hyperparameters, we first set the learning rate to a magnitude of 10^{-3} by pre-experimentation and then compared between $\{1, 2.5, 5\} \times 10^{-3}$ to determine a learning rate of 0.0025. We select SGD as the optimiser with a momentum of 0.9 and weight decay of 0.0001. The learning rate is decayed at epochs 16 and 22. We set the maximum epoch to 24 and implement the early stopping technique based on the performance of the validation set. We also implement a linear warm-up technique for the first 500 iterations of training with a warm-up ratio of 0.001.

We conduct comparison experiments among SRAF-Net [15], Faster-RCNN-FPN, SE-Net, CBAM and BCA-Net. SFAR-Net is a model designed for unclassified dumpsites, with a deformable convolution module to better extract features of irregularly shaped objects. SRAF-Net and BCA-Net optimise the model from two different perspectives for extracting features of dumpsites, so the two detectors are compared here. Faster-RCNN-FPN without any attention module is employed here to reflect the impact of attention mechanisms on dumpsite detection. Additionally, the Faster-RCNN-FPN here does not utilise Focal Loss [16] for a fair comparison on the long-tailed dataset. Experiments on Focal Loss are presented in the experiments addressing the long-tail problem (Supplementary Table 5).

Experiment on unclassified dumpsite dataset

Before detecting the classified dumpsites, we also perform a detection test on the unclassified dumpsite dataset in the form of [17, 18, 19]. It is necessary to conduct this performance test to verify that the "dumpsite" as a whole can be well distinguished by the model, which is an essential step for fine-grained detection. If the "dumpsite" as a whole cannot be detected, let alone the classified dataset.

Supplementary Table 1: Results of the five models on dumpsite test set without category labels.

Model	Average Precsion	Epoch
SRAF-Net	69.72% (best)	22th
Faster-RCNN-FPN	76.88% (best)	13th
SE-Net	78.91% (best)	19th
CBAM	79.03% (best)	24th
BCA-Net	80.73%	$\mathbf{12^{th}}$
**BCA-Net**	**81.32%(best)**	21th

To achieve this, we first set all the object categories in our dataset as "dumpsite". After that, 80% of the image tiles are divided into the training set (including 60% train set and 20% validation set) and the other 20% into the test set. Supplementary Table 1 shows the test set results of SRAF-Net, Faster-RCNN-FPN, SE-Net, CBAM and BCA-Net on the unclassified dataset, where the label "(best)" refers to the best result out of 24 epochs, and the "Epoch" column represents the epoch in which the best result occurs. In terms of performance, BCA-Net outperforms deformable convolution-based SRAF-Net by 11.6% and outperforms Faster-RCNN-FPN, which does not use an attention mechanism, by 4.44%. Compared to the widespread attention mechanism approach, BCA-Net outperforms SE-Net and CBAM by 2.41% and 2.29% on the unclassified dumpsite dataset. These results demonstrate that BCA-Net can extract the features of dumpsites and identify them better than other models. It is also noteworthy that BCA-Net achieves 80.73% AP after 12 epochs. In other words, BCA-Net can converge faster with less learning process than other approaches, which indicates that BCA-Net is more effective in feature extraction than other models.

In order to compare the inference results of several models with different architectures more intuitively, we present the detection results of three approaches (SRAF-Net based on deformable convolution, Faster-RCNN-FPN without attention mechanism, and BCA-Net leveraging attention mechanism) on some test set images. Some typical inference results are presented in Supplementary Figure 1. Columns (a) to (d) represent the ground truth, predicted result of SRAF-Net, Faster-RCNN-FPN, and BCA-Net, respectively. By comparing the performance of the three models on typical samples, four facts can be summarized: (1) In the results of the first row, the boundaries of the Bounding Box (red) predicted by the BCA-Net is closer to the ground truth (green) than the other two models, which means a better convergence of the boundary information. (2) In the second row, Faster-R-CNN-FPN and the previous method SRAF-Net cannot detect all dumpsites in the given tile. In contrast, BCA-Net does well in finding these "difficult" samples (small sized samples with similar characteristics to the background); (3) In the third row, the results of Faster-RCNN-FPN and SRAF-Net have false alarms individually, while BCA-Net only

detects the real dumpsites. (4) However, none of the three models can successfully predict dumpsite in the last row. This dumpsite data may have fewer uniform features in morphology than common dumpsites, which conveys that deep neural networks models cannot give competitive results for abnormal points.

Experimental results on classified dumpsite dataset

After verifying the performance of the models on the unclassified dumpsite dataset, we explore the performance of these models on the classified dumpsites in this section. Due to the significant long-tail distribution in the dataset, this section contains comparisons between models as well as that of different techniques on the "tailed" class.

Supplementary Table 2: Results of the five models on classified dumpsite test set without using training strategies (data augmentation and category balancing). Domestic Waste, Construction Waste, Agricultural Waste and Covered Waste is abbreviated as DW, CW, AW and CW2.

Model	AP				mAP	AP_{Medium}	AP_{Large}
	DW	CW	AW	CW2			
SRAF-Net	60.07%	39.96%	34.81%	**7.64%**	35.62%	44.72%	39.51%
Faster-RCNN-FPN	65.96%	50.63%	37.62%	0.23%	38.61%	55.06%	42.76%
SE-Net	67.58%	54.57%	39.00%	0.31%	40.37%	58.26%	45.01%
CBAM	64.78%	59.46%	38.73%	0.47%	40.86%	57.97%	43.33%
**BCA-Net**	**68.91%**	**60.06%**	**41.29%**	0.58%	**42.71%**	**59.16%**	**48.53%**

Supplementary Table 2 compares the five models without any training strategies (data augmentation and category balancing). Considering that the "tailed" class significantly impacts mAP, we added AP_{Medium} and AP_{Large} evaluation metrics in Supplementary Table 2 and 3. Without any training strategy, BCA-Net performs better than the other four models on DW, CW and AW. For CW2 with a tiny amount, the APs of most models are close to 0. SRAF-Net, which uses Focal Loss, performs relatively better on CW2. However, the performance of SRAF-Net on the other three dumpsites is significantly lower than that of the other four models due to the effect of Focal Loss on the parameter space. AP_{Medium} and AP_{Large} in Supplementary Table 2 are more indicative of the overall detection capability of several models for the classified dumpsite dataset. Similarly, BCA-Net scores better than the other models on AP_{Medium} and AP_{Large}. Supplementary Table 2 illustrates that BCA-Net is better at detecting "non-tailed" classes without any training strategy. For the "tailed" class, all models need better solutions to improve performance. Inspired by [20], we try to find the reasons why models fail to detect dumpsites. By observing all the CW2 detection results in the test set, we find that all the reasons that models cannot detect CW2 are missed detections. Specifically, all the proposals around the covered waste are with very low confidence values, which means these proposals could not be considered as bounding boxes afterwards. We think that the model has too few chances to learn CW2 and thus cannot extract enough features of CW2. Therefore, we hypothesise that this result would be improved by increasing the number of times the model learns CW2, and we conduct relative experiments below.

Supplementary Table 3: Results of the five models on classified dumpsite test set with two training strategies (data augmentation and category balancing).

Model	AP				mAP	AP_{Medium}	AP_{Large}
	DW	CW	AW	CW2			
SRAF-Net	57.14%	37.48%	39.88%	77.93%	53.10%	40.56%	36.51%
Faster-RCNN-FPN	66.71%	52.64%	43.29%	83.78%	61.61%	53.73%	41.15%
SE-Net	64.18%	58.79%	53.01%	93.21%	67.30%	56.09%	43.27%
CBAM	65.90%	57.37%	44.14%	94.43%	65.46%	55.81%	42.17%
**BCA-Net**	**67.95%**	**59.33%**	**55.79%**	**96.67%**	**70.09%**	**58.72%**	**46.73%**

Supplementary Table 3 compares five models employing two training strategies. Macroscopically, BCA-Net achieves better performance in all metrics. For "tailed" CW2, training strategies increase AP_{CW2} to $77 \sim 96\%$ by raising the number of samples or the number of times models learn CW2. The significant improvement of AP_{CW2} reflects two facts. On the one hand, CW2 is so distinguishing that models can identify it well as soon as they thoroughly learn CW2's features. On the other hand, the experiments confirm the significant influence of the

long-tail distribution on models. There is an interesting phenomenon in Supplementary Table 3. With the use of training strategies, the performance of AW and CW2, which have a smaller number of samples, improves significantly compared to Supplementary Table 2, while the performance of DW and CW, which have larger numbers of samples, decreases slightly. We speculate that the training strategies change the original distribution of the sample space, resulting in a reduction in the number of learning times for DW and CW. To further explore the impact of the two training strategies on the four categories of dumpsites, we conduct the experiments in Supplementary Table 4.

Supplementary Table 4: The performance of BCA-Net on the four types of dumpsites using different training strategies.

Model	Data Augmentation	Category Balancing	AP DW	AP CW	AP AW	AP CW2	mAP
BCA-Net	✗	✗	**68.91%**	**60.06%**	41.29%	0.58%	42.71%
BCA-Net	✓	✗	64.67%	51.14%	50.14%	12.14%	44.52%
BCA-Net	✗	✓	62.14%	51.12%	44.83%	60.25%	54.58%
**BCA-Net**	✔	✔	67.95%	59.33%	**55.79%**	**96.67%**	**70.09%**

We conduct ablation experiments on the best-performing models in Supplementary Table 3 for "data augmentation" and "category balancing". At a macro level, the model utilising both ticks performs well on four classes. In detail, the addition of any training strategy reduces the model's ability to detect DW and CW, while simultaneous use of both training strategies reduces this lousy effect. Furthermore, "data enhancement" is better for AW with a higher amount than "category balancing". For CW2 with an extremely small amount, category balancing is much more effective than data augmentation.

Supplementary Table 5: Comparisons between Focal Loss and training strategies (data augmentation and category balancing).

Model	Focal Loss	Two Strategies	AP DW	AP CW	AP AW	AP CW2	mAP
BCA-Net	✗	✗	68.91%	60.06%	41.29%	0.58%	42.71%
BCA-Net	✓	✗	67.6%	56.6%	33.7%	5.78%	40.92%
BCA-Net	✗	✓	67.95%	59.33%	55.79%	96.67%	70.09%
**BCA-Net**	✔	✔	63.02%	57.91%	44.15%	66.79%	57.97%

In addition to solving the long-tail problem by resampling, we also try to investigate the improvement of the "tailed" class by a reweighting scheme. We implement reweighting by adding Focal Loss to BCA-Net. Supplementary Table 5 illustrates the impact of both ideas on the performance of BCA-Net. Without training strategies, Focal Loss slightly improves BCA-Net's understanding of CW2. Actually, Focal Loss is overweighted in favour of CW2 when calculating the loss as the sample size of CW2 is too small, which results in lower performance for other classes. When training strategies are used, Focal Loss also limits BCA-Net's ability to detect all four types of dumps.

Similar to Supplementary Figure 1, Supplementary Figure 2 shows the inference results for several test set images (with two training strategies). (a) to (d) represent the ground truth, SRAF-Net, Faster-RCNN-FPN, and BCA-Net after using the two techniques, respectively. The results in the first row show the detection of long-tailed classes by the three models. It can be seen that BCA-Net accurately detects the boundaries of the dumpsite and even surpasses manual annotation in detail while the other two have all or part of the missed detection. The second row shows the detection of the three models for the "difficult" samples in the "non-tailed" class, and the results show that BCA-Net correctly predicts the boundaries and categories of the sample. The misjudgment and the false alarms of the first two models are shown in the last two rows, respectively. The comparison of the detection results on four typical samples shows that BCA-Net with the two techniques can achieve better performance.

In fact, although BCA-Net performs the best among the three models, dumpsite classification in satellite images still needs some enhancement before being used in practice, including the limitation of image resolutions and indistinguishable dumpsite categories as mentioned earlier in this section. By comparing the results of the unclassified section and this section, the mAP of all categories on the classified dataset is still far lower than the AP of the unclassified dumpsite dataset.

Insights through the BCA-Net

The experiments in the first two sections demonstrate that BCA-Net performs relatively well and has the potential to replace humans in the task of dumpsite detection. To visually explain why BCA-Net can produce better results, we use the Grad CAM [21] technique to peer into the feature encoder. Specifically, this technique allows us to see what the model is focusing on. Supplementary Figure 3 presents the visualisation of the feature layers generated from the trained BCA-Net and the Faster-RCNN-FPN during the inference process. The different brightnesses of these regions in pictures represent different levels of attention paid by models, and the higher the brightness of the regions, the higher the model's attention. The effect of the BCA module can be visualized with the Grad CAM.

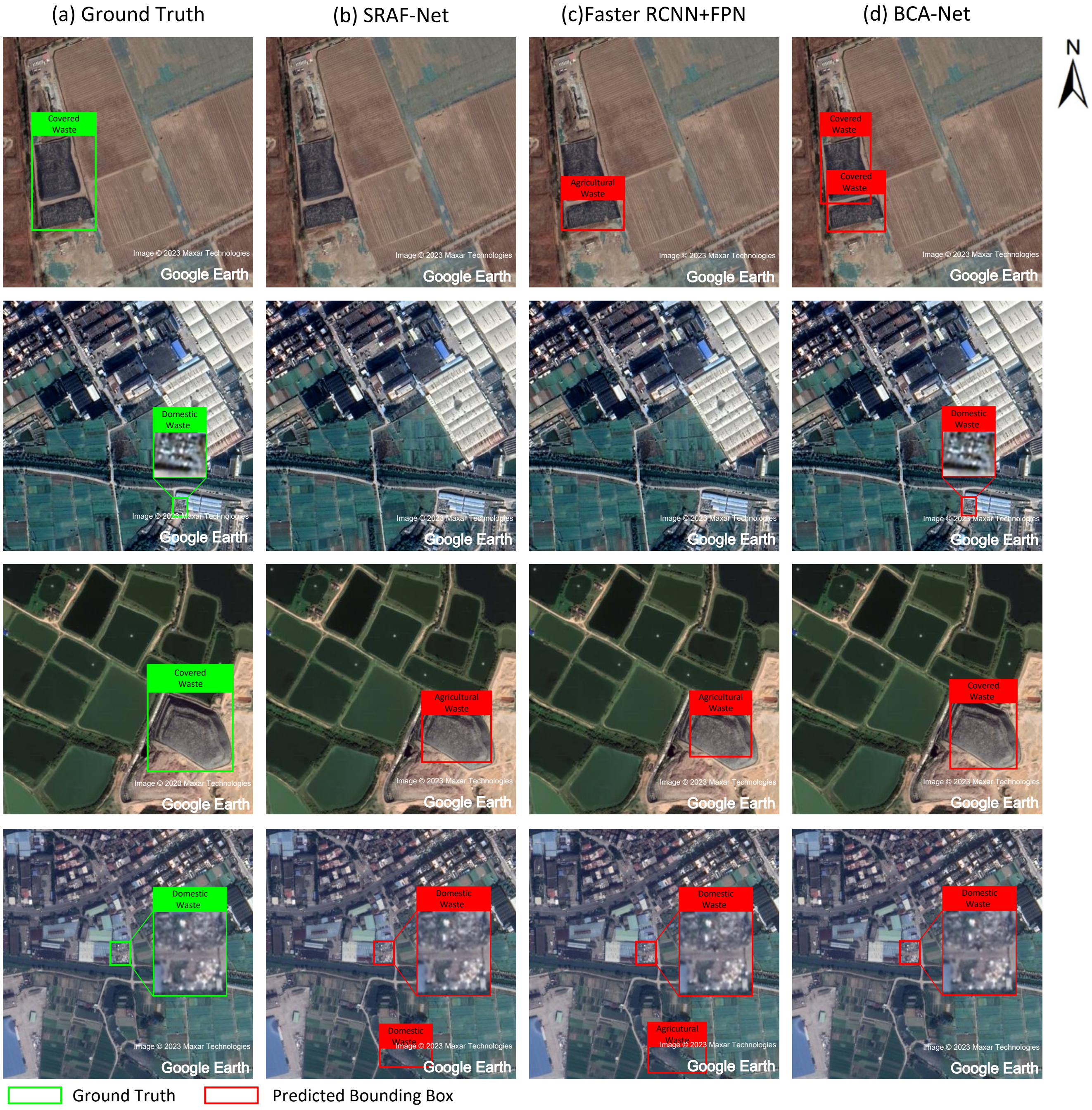

Supplementary Figure 2: (a) Ground truth of four examples. (b) Predicted results of SRAF-Net. (c) Predicted results of Faster-RCNN-FPN. (d) Predicted results of BCA-Net. All of the above models incorporate two strategies when training, and all these images are from the test set.

Supplementary Figure 3 shows the output results of the four residual blocks (C2 to C5) in the backbone network ResNet50 and the P2 to P5 layers of the Neck partial pyramid structure (trained with two training strategies). The model structures shown in Supplementary Figure 3 can be positioned in Figure 6 (body part) and the test sample selected here for inference is the tile (1024*1024 pixels) presented in the first row of Supplementary Figure 2. For layers C2 and C3, there is little difference in the distribution of feature attention between the two models on the test samples, essentially because the structure of the two models here is the same. As mentioned earlier, the BCA module replaces the C4 and C5 modules in the Faster-RCNN-FPN. Supplementary Figure 3 tells us that the two models start to differ from the C4 and C5 levels. Specifically, the model with the BCA module starts to focus on the "covered waste" location area in C4, while C4 in the Faster-RCNN-FPN focuses on almost the same area as C3, ignoring the main area with "covered waste". More importantly, the two models extract significantly different results in the C5 feature layer. All regions belonging to the C5 layer in the Faster-RCNN-FPN have almost the same brightness, and the model's attention cannot be focused on the locations containing dumpsites. With the addition of the BCA module, the model pays more attention to most of the areas containing the dumpsite, with the other areas receiving considerably less attention compared to the Faster-RCNN-FPN.

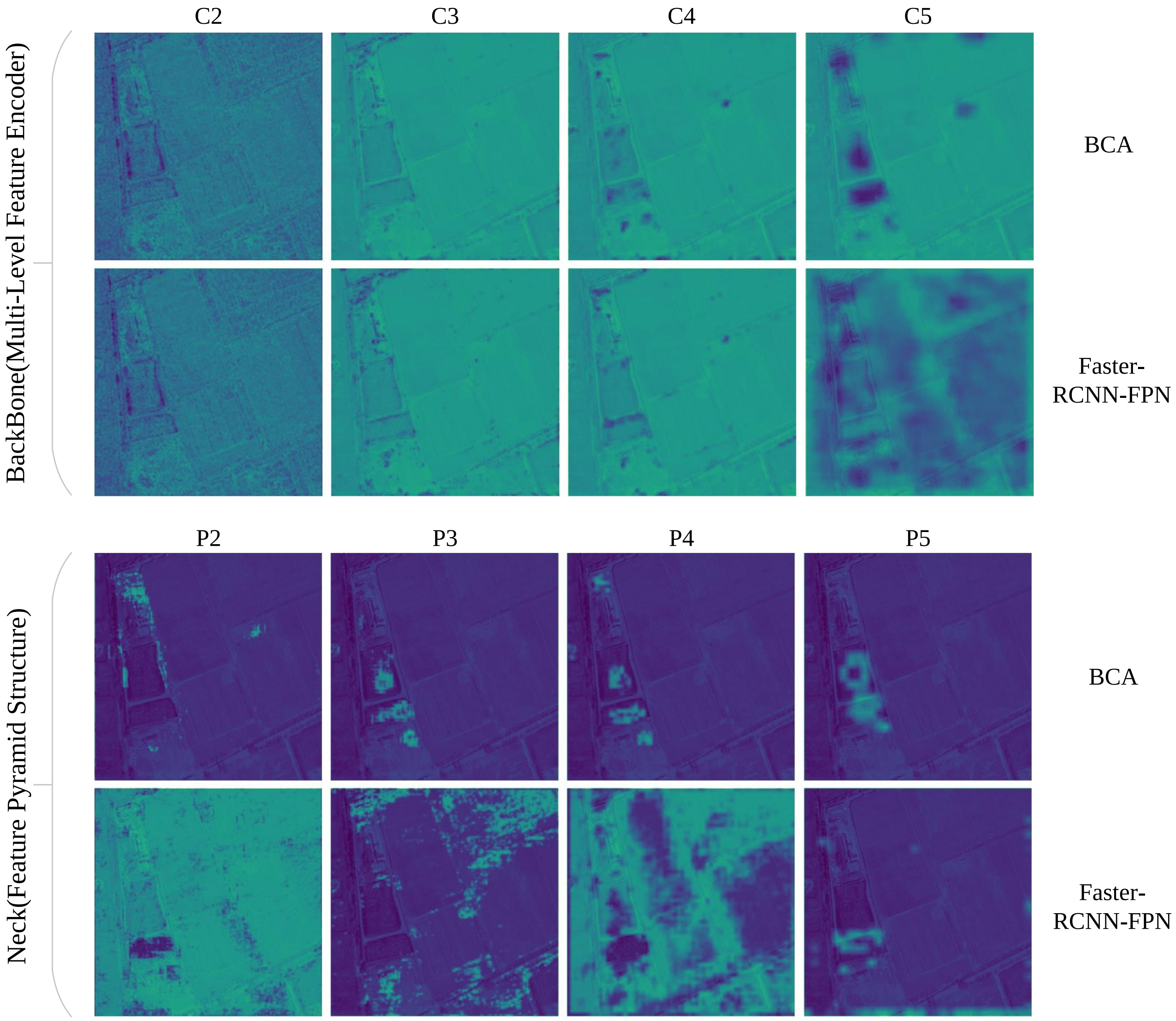

Supplementary Figure 3: Visualization results of BCA-Net, including the intermediate feature layers of backbone and neck.

From Figure 6 (body part), the superposition of P2 $\sim$ P5 is finally sent to the regressor and classifier to determine the location and category, so the attention of P2 $\sim$ P5 layers is more important. From Supplementary Figure 3, the focus area of the Faster-RCNN-FPN's P2 and P5 is close to its C5, suggesting that the error information provided by C5 was amplified in the follow-up process, which further results in the model being unable to determine the location and class information of possible objects. Even though the P5 layer of the Faster-RCNN-FPN model obtained partially correct information through several training iterations, the first column (c) of Supplementary Figure 2 shows that the final inference result of the Faster-RCNN-FPN is not only incomplete in terms of position, but also incorrect in terms of category. However, the model with the BCA module pays attention to the features containing dumpsite in (P3 $\sim$ P5) and completely removes the noisy features in P5. From the results in the first column (d) of Supplementary Figure 2, the area focused by P5 is almost the area of object. At the same time, the model's judgement of the category is correct due to the complete extraction of the features containing objects.

Through the visualization process in Supplementary Figure 3, it can be concluded that the addition of BCA modules achieves essential improvements in extracting high-level features. At the same time, it shows that the design of the network structure has a vital influence on the extraction of core information in the dataset. The starting point for designing BCA is to enhance the feature extractor's ability to focus on crucial object information in the feature extracting process, and the visualization results confirm that the addition of the BCA module has reached the expected effect.

Supplementary Discussion

The effects of the hyperparameter α

Supplementary Table 6: Ablation experiment results of the hyperparameter α, including memory and time consumption.

Model	α	AP				mAP	GPU Memory	Time (One Epoch)
		DW	CW	AW	CW2			
BCA-Net	32	62.56%	57.21%	54.31%	95.20%	67.32%	**6795MB**	**7.0 Min**
BCA-Net	16	67.95%	59.33%	**55.79%**	96.67%	70.09%	**7515MB**	**7.5 Min**
BCA-Net	8	**70.53%**	**65.99%**	51.44%	**97.90%**	**71.47%**	11320MB	8.1 Min

As mentioned in Supplementary Method, the BCA module divides the feature layers of $C \times H \times W$ into $(C \times H \times W)/\alpha^2$ blocks in the blocking process, and the selection of hyperparameter α here has a significant impact on model performance. Theoretically, the more blocks are separated, the better the model can classify the extracted fine-grained feature information according to different degrees of importance. In practice, more blocks mean greater memory consumption and training time, and if the number of blocks reaches a certain level, the model may not be able to run on personal devices such as laptops. Three cases are tested here, and α is set as 32, 16, 8 respectively (H and W in ResNet50 are both powers of 2, so here considering the divisibility, α is also set to the power of 2). Supplementary Table 6 shows the performance, inference time, and memory consumption of each model under the three values of α, and the graphics device used here are NVIDIA RTX 3090 GPUs.

It is worth mentioning that when α varies from 16 to 8, GPU consumption increases by about 51% with only 1.45% growth of the mAP. Considering the memory consumption, we set α to 16 in the previous experiments. The trade-off between memory consumption and model performance is an essential issue in DCNN constructures through the above comparison.

Impact of resolutions on model inference

As the dataset (classified) consists of images with multiple resolutions, we conducted a simple statistic on the detection performance of objects with different resolutions in the test set. Supplementary Table 7 tells us that there is a significant difference in the recognition ability of the model for images of different resolutions and that the higher the resolution, the better the recognition.

Supplementary Table 7: Results of the models on test sets with different resolutions. (Given the classified dataset's size and actual distribution, we train models with data of all resolutions)

	$AP_{0.3m}$ (22.9%)	$AP_{0.6m}$ (62.3%)	$AP_{1.0m}$ (14.7%)
SRAF-Net	57.39%	39.43%	31.13%
Faster-RCNN-FPN	64.04%	59.23%	45.15%
SE-Net	66.68%	60.04%	48.91%
CBAM	65.08%	61.24%	47.77%
**BCA-Net**	**69.35%**	**62.97%**	**51.24%**

The strengths and weaknesses of BCA-Net

During experimenting, we first apply the model to dumpsite data without category labels. In other words, all dumpsites have only one type of label, "dumpsite". Due to the existence of the BCA module, the model's performance is effectively improved compared to the Faster-R-CNN-FPN structure. In the visualization process, it can be found that compared with the Faster-R-CNN-FPN structure, the addition of BCA enables the model to extract more detailed dumpsite features and better focus on the characteristics of the dumpsite. However, BCA-Net follows the anchor-based method, generating many pre-set anchors to generate candidate bounding boxes before determining the object candidate area. This approach will bring two effects. Firstly, these anchors can ensure that the model can capture those areas containing dumpsite objects as much as possible. On the other hand, this method will analyse many areas that do not have dumpsite, which will significantly increase the false alarm rate of inference results. The anchor-free method is followed in SRAF-Net, but it does not bring perfect experimental effects. Therefore, high false alarm rates may become the next focus of the dumpsite detection.

Supplementary Notes

Design of the Global Dumpsite Index (GDI)

The original idea of the Global Dumpsite Index is to use a number to indicate the level of regional dumpsite numbers on a global scale. The simple utilization of the total number of dumpsites directly will be exponentially distributed in ascending order. This could lead to the conclusion that the index differs several times between two cities within the same level. As a result, we employ a simple but effective form of logarithm for a linearly distributed index across the 28 cities. We also divide the 28 cities into three classes based on the proposed Global Dumpsite Index, which is designed to simplify the comparison process for subsequent researchers. Specifically, the GDI is calculated as follows:

$$GDI = \log_2 N,$$

where N denotes the number of dumpsites.

Fieldwork

In order to ensure the correctness of the labelling process, we implemented extensive field research on the dumpsites. Between December 2020 and May 2021, we visited more than 500 dumpsites and took many photographs. We visited 91 cities in China, including Beijing, Shanghai, Tianjin, Hefei, etc. as shown in the Supplementary Table 8. For each city, we spent around one week to verify dumpsites. For dumps out of China, we have searched help from scholars in Germany and Japan to identify. Due to COVID-19, we could not access all the dumpsites. Still, we tried our best to identify some hard-to-reach dumps through Google Street View and other online resources. Supplementary Figures 4 and 5 show part of field photos with their corresponding satellite images.

Supplementary Table 8: Detailed information on fieldwork, including cities, duration, frequency, date and method.

Province	Cities	Duration	Frequency	Date	Method
Beijing	Beijing	3.5 days	7	January 2021	Visits & Photos
Shanghai	Shanghai	7 days	31	January 2021	Visits & Photos
Tianjin	Tianjin	7 days	28	January 2021	Visits & Photos
Anhui	Heifei, Bengbu, Huainan, Huaibei, Tongling, Anqing<br>Chuzhou, Fuyang, Suzhou, Lu'an, Bozhou, Xuancheng	7 days per city	66	February 2021	Visits & Photos
Fujian	Fuzhou, Putian, Sanming, Quanzhou, Zhangping, Nanping<br>Longyan, Ningde	7 days per city	34	December 2020 to January 2021	Visits & Photos
Guangdong	Guangzhou, Shaoguan, Shenzhen, Zhuhai, Shantou, Jiangmen<br>Zhanjiang, Maoming, Zhaoqing, Huizhou, Meizhou, Heyuan<br>Yangjiang, Qingyuan, Dongguan, Zhongshan, Chaozhou, Yunfu	7 days per city	76	January 2021 to March 2021	Visits & Photos
Hainan	Sanya, Wuzhishan, Qionghai, Danzhou, Wenchang, Wanning, Dongfang	7 days per city	23	March 2021	Visits & Photos
Jilin	Changchun, Jilin, Siping, Liaoyuan, Tonghua, Baishan<br>Songyuan, Baicheng, Yanbian	7 days per city	44	April 2021	Visits & Photos
Neimenggu	Huhehaote, Baotou, Chifeng, Tongliao, Eerduosi, Hulunbeier<br>Bayannaoer, Xing'an, Xilinguole	7 days per city	53	April 2021	Visits & Photos
Qinghai	Xining, Haidong, Tongren, Jianzha, Zeku	7 days per city	29	December 2020	Visits & Photos
Shandong	Qingdao, Zibo, Zaozhuang, Weifang, Jining, Rizhao<br>Linyi, Dezhou, Binzhou	7 days per city	44	May 2021	Visits & Photos
Shanxi	Taiyuan, Datong, Yangquan, Changzhi, Jincheng, Shuozhou<br>Jinzhong, Yuncheng, Yizhou, Linfen, Lvliang	7 days per city	67	May 2021	Visits & Photos

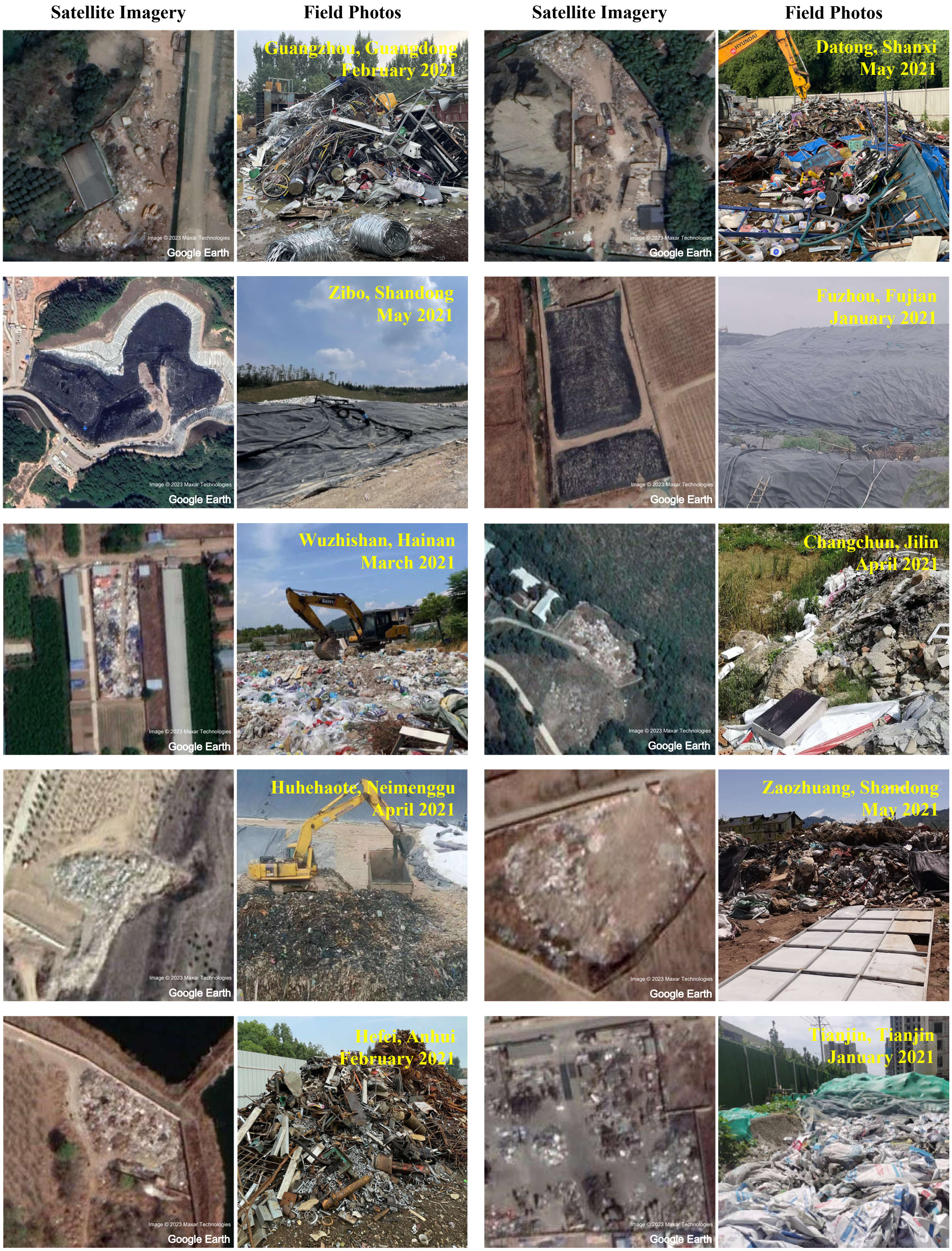

Supplementary Figure 4: Field photographs and corresponding satellite images of some dumpsites. (Part 1)

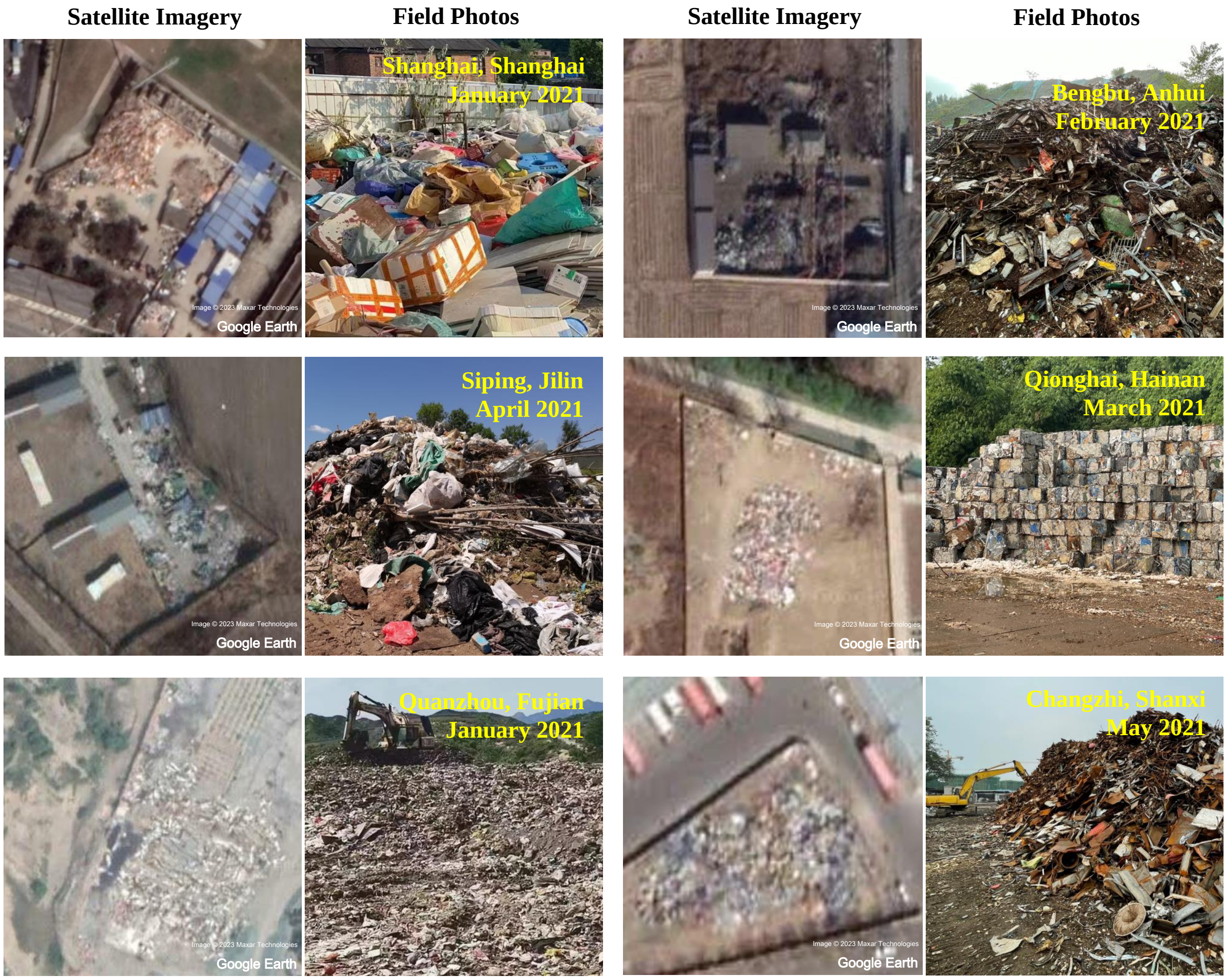

Supplementary Figure 5: Field photographs and corresponding satellite images of some dumpsites. (Part 2)

Annotation

Image Collection. The satellite imagery included in the dataset is from the Gaofen [22] and SuperView [23] series of satellites. We collected about 7000 km^2 of satellite images to construct the dataset based on the selected area, containing no duplicate images. We cut the images into multiple 1024 × 1024 tiles. Considering that some objects may exist at the edges of the tiles, we set the step size to 512 instead of 1024 when cutting.

Category Selection. There are various types of dumpsites defined, including but not limited to "recyclable/non-recyclable waste", "food waste", "industrial waste", "domestic waste", "construction waste", "agricultural waste", etc. In this work, some of the types above can be distinguished in the satellite images, while others not. Therefore, we classify the dumps according to distinguishability, appearance, origin, form of disposal and past work. Previous work [24] classified dumpsites into domestic waste and construction waste when implementing satellite imagery for dump detection in South Africa, so our dataset also contains both categories. Recent works [7, 8, 9] demonstrate the effectiveness of high density polyethene (HDPE) films in suppressing harmful gases and permeate from landfills. Considering the significance of this low-cost and efficient method for dumpsite treatment, we classify this type of dumpsite in the satellite images as "covered waste". In addition, the literature on agricultural waste management has been increasing recently [25], and the disposal of agricultural waste generally requires special treatment and reuse [25, 26, 27]. Agricultural waste can also be distinguished by its appearance in satellite images, so we have included this category in the dataset.

Annotation Procedure. The process of dumpsite annotation is different from the annotation of most datasets (e.g. COCO, Pascal VOC and DOTA datasets). The main difference is that the presence of dumpsites on satellite images is unpredictable. In other words, most images do not contain dumpsites, and it is difficult to determine which city area has a large number of dumpsites. We think areas with large population and poor environmental performance will likely have more dumpsites, so we select seven developing countries to create the dataset based on population size and the Environmental Performance Index (EPI). Our annotation team consists of 12 experts in the field of remote sensing. Before annotation, all members participate in a field research workshop and identify four kinds of dumpsite characteristics for harmonisation (Figure 1d in the body part). The dumpsite annotation is divided into three stages: category-free filtering, category labelling and bounding box annotation.

Category-free filtering is the process of sifting through the tiles of a large number of satellite images that contain any dumpsite. No distinction is made between categories at this stage. Three annotators judge each tile to ensure the reliability of the results, and a tile is deleted only if all three annotators agree at the same time that it does not contain a dumpsite. In addition, due to the cutting step size of 512, many duplicate dumpsites appear in the results. However, as the tile numbers are in the same order as the cut, the duplicate piles are all numbered closely. All annotators remove the duplicate dumpsites before submitting the results.

After the previous stage, the remaining tiles do not duplicate each other and have the potential to contain dumpsites. This stage involves labelling categories, i.e. each tile is labelled with possible categories. The results of this stage will be three scenarios: no dumps, single category and multiple categories. The annotators in this stage need to be clear about each type of dump, so six annotators are selected for the test to annotate the categories. The test procedure is as follows: all annotators are asked to classify a sample of 50 dumps after learning, and the test is passed only if all samples are correctly classified. The six annotators that pass the test the fastest perform this annotation phase. Three selected annotators label each tile, and when there is a disagreement between the three annotators, a discussion is held to determine the category.

All the tiles containing dumpsites are labelled with categories. At this stage, each annotator only has to annotate one category of the bounding box, which also decreases the annotation difficulty considerably. All tiles are divided into four clusters according to the category of dumps they contain, and tiles containing more than one category are annotated in multiple rounds according to the number of categories. Annotators are divided into four groups, with each group (three annotators) responsible for annotating one category. As before, the results are determined by discussion within the group when tiles within the same group are ambiguous.

Annotation Verification. To validate these annotations, we have carried out extensive fieldwork to visit part of dumpsites in our dataset. We visited over 500 of the 2,500 dumpsites, with a sampling rate of 20%. All these sampled dumpsites are confirmed to be authentic dumps. The Fieldwork section illustrates the detailed information of our massive visits.

Through these complex and rigorous annotation processes, the quality of our annotations can be better than a single, even several untrained remote sensing experts.

Detection Tools.

We have published our dataset and code. In addition, we have provided an environment that can be run directly. This environment allows the user to ignore the code details and focus more on dumpsite detection. Supplementary Figure 14 is an introduction to the tool.

Supplementary Figures

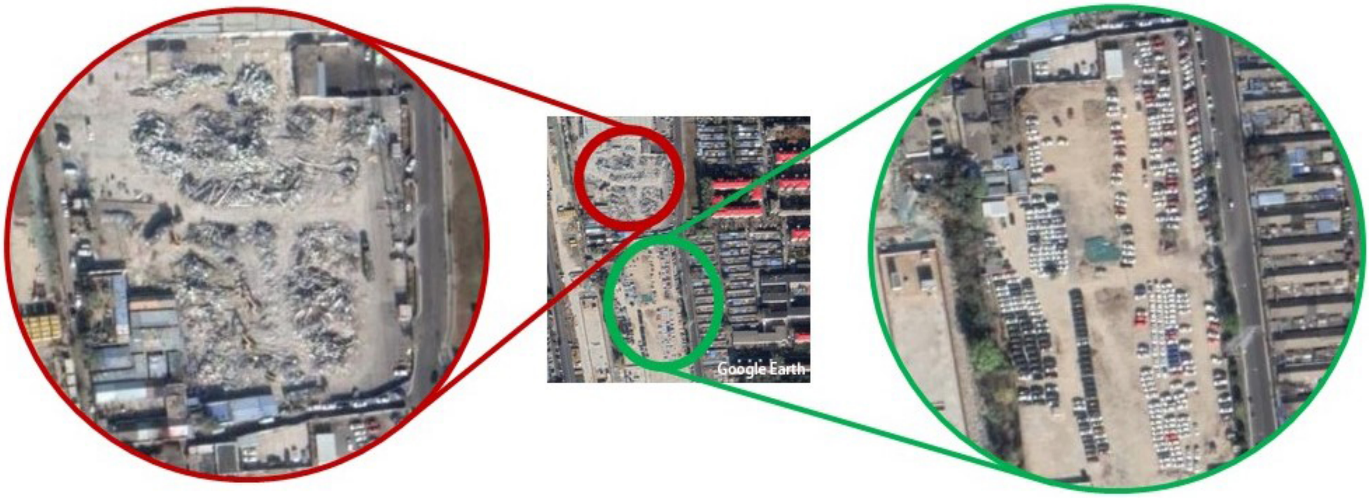

Supplementary Figure 6: Zoomed-in view of the two highlighted areas of Figure 2b (main body).

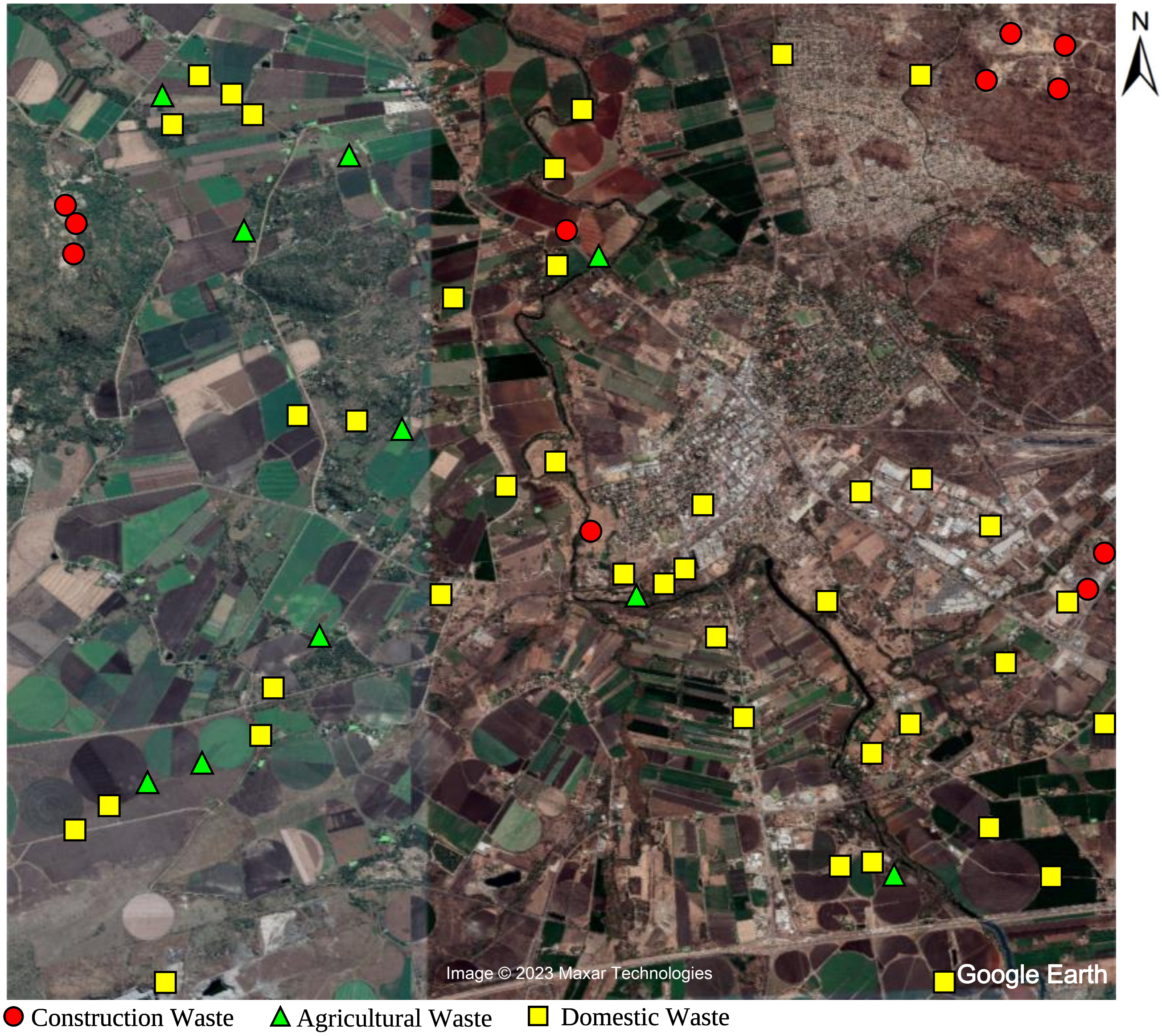

Supplementary Figure 7: Dumpsite distribution in parts of Bojanala.

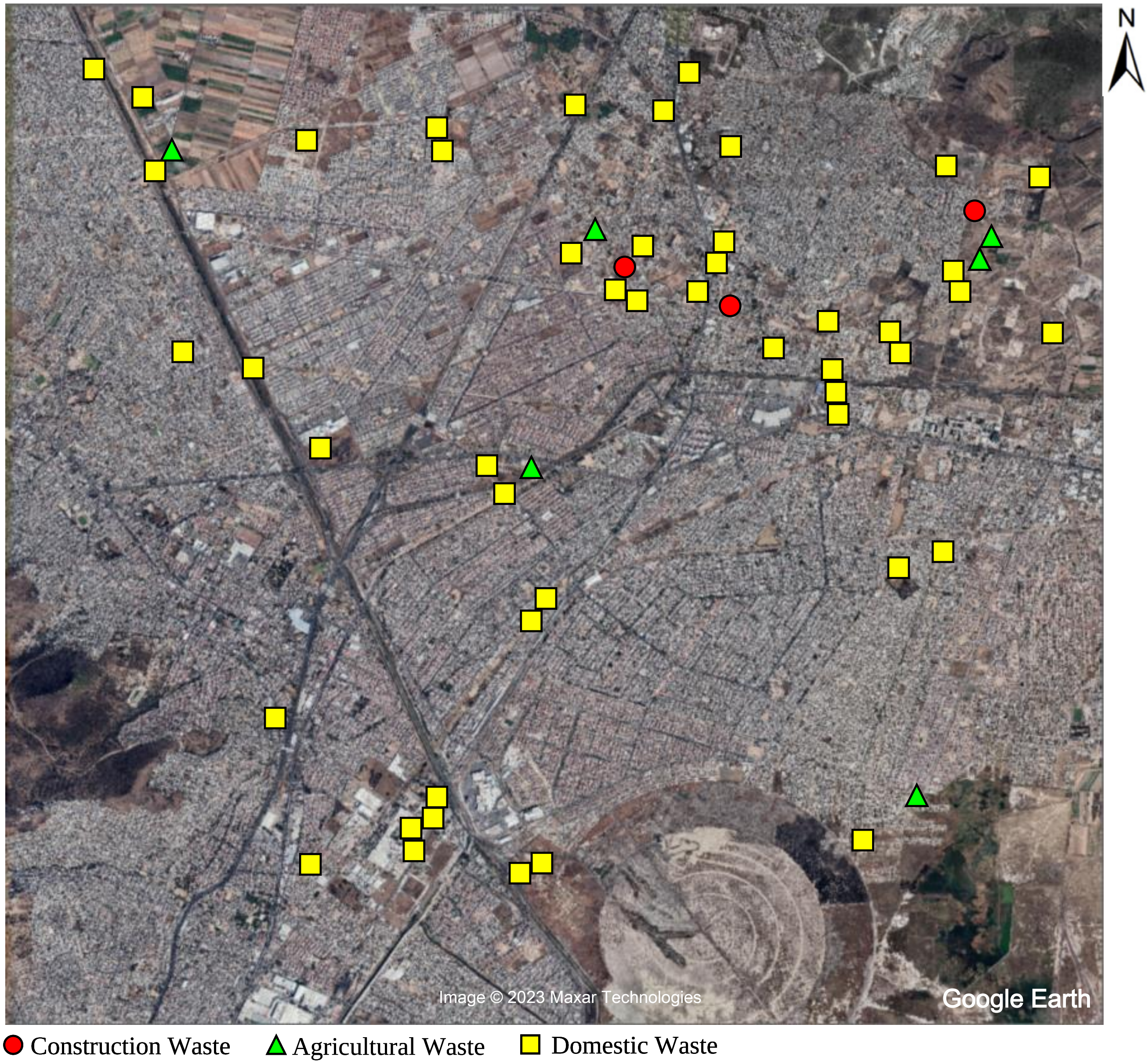

Supplementary Figure 8: Dumpsite distribution in parts of Ecatepec.

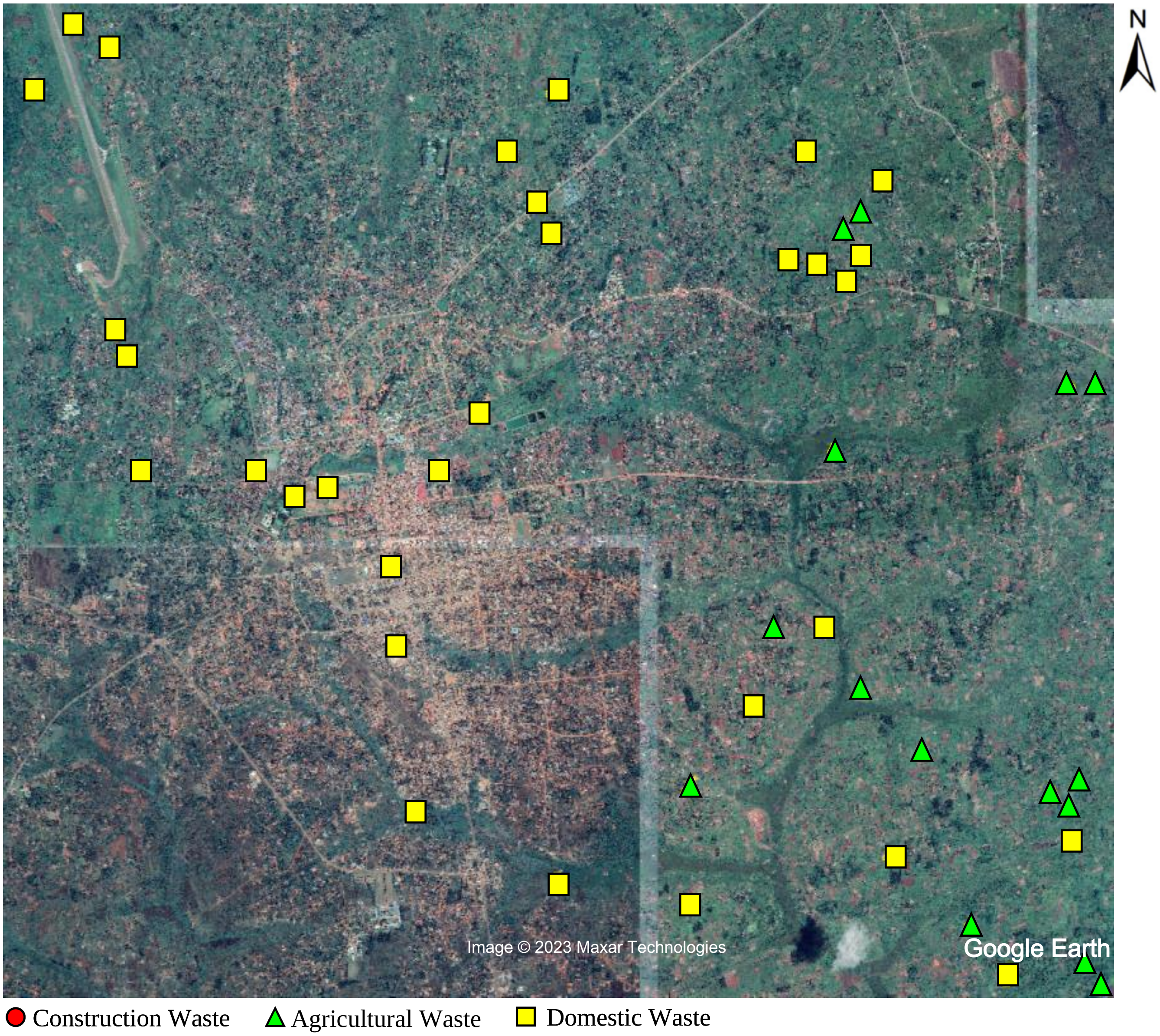

Supplementary Figure 9: Dumpsite distribution in parts of Gulu.

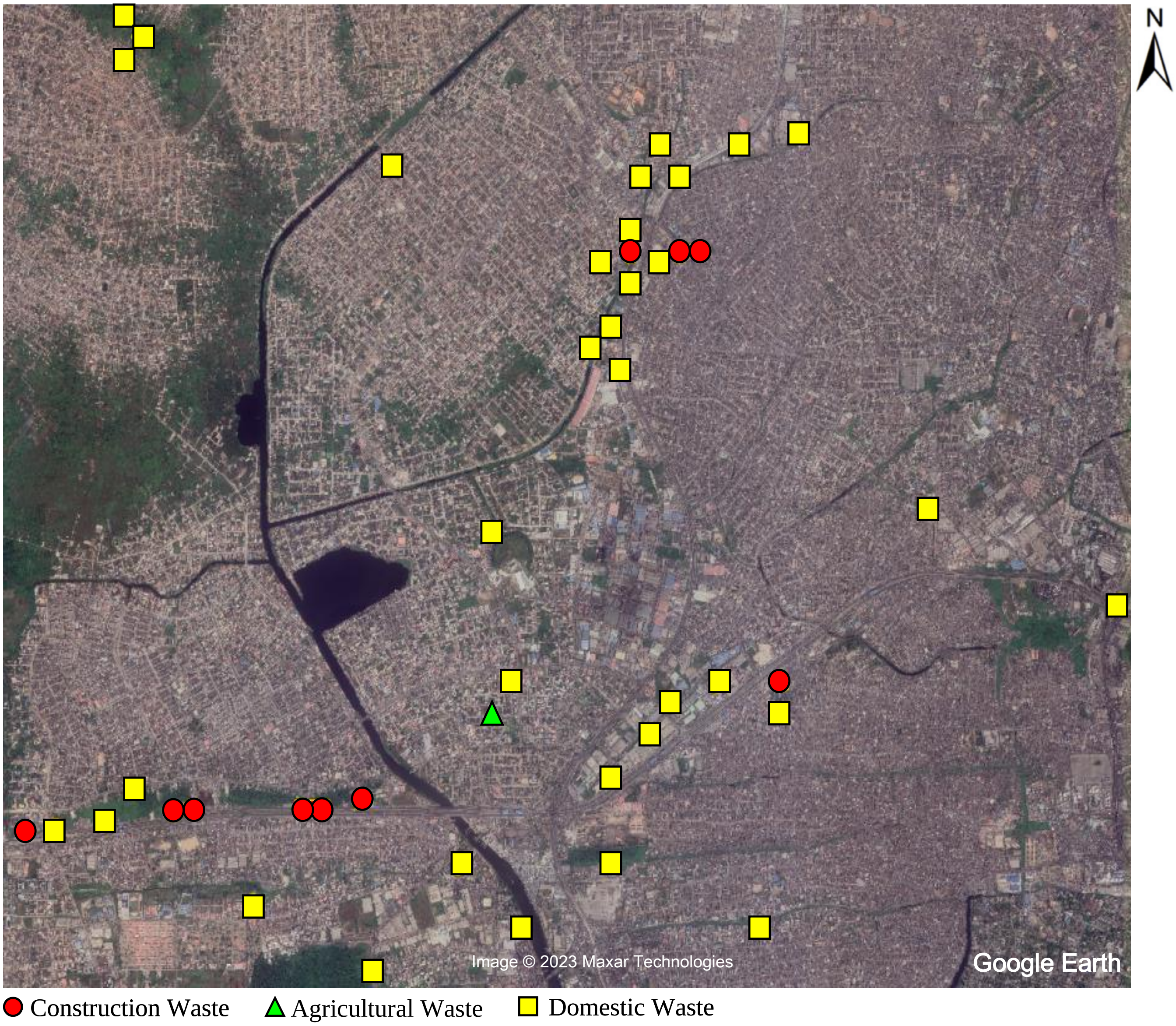

Supplementary Figure 10: Dumpsite distribution in parts of Lagos.

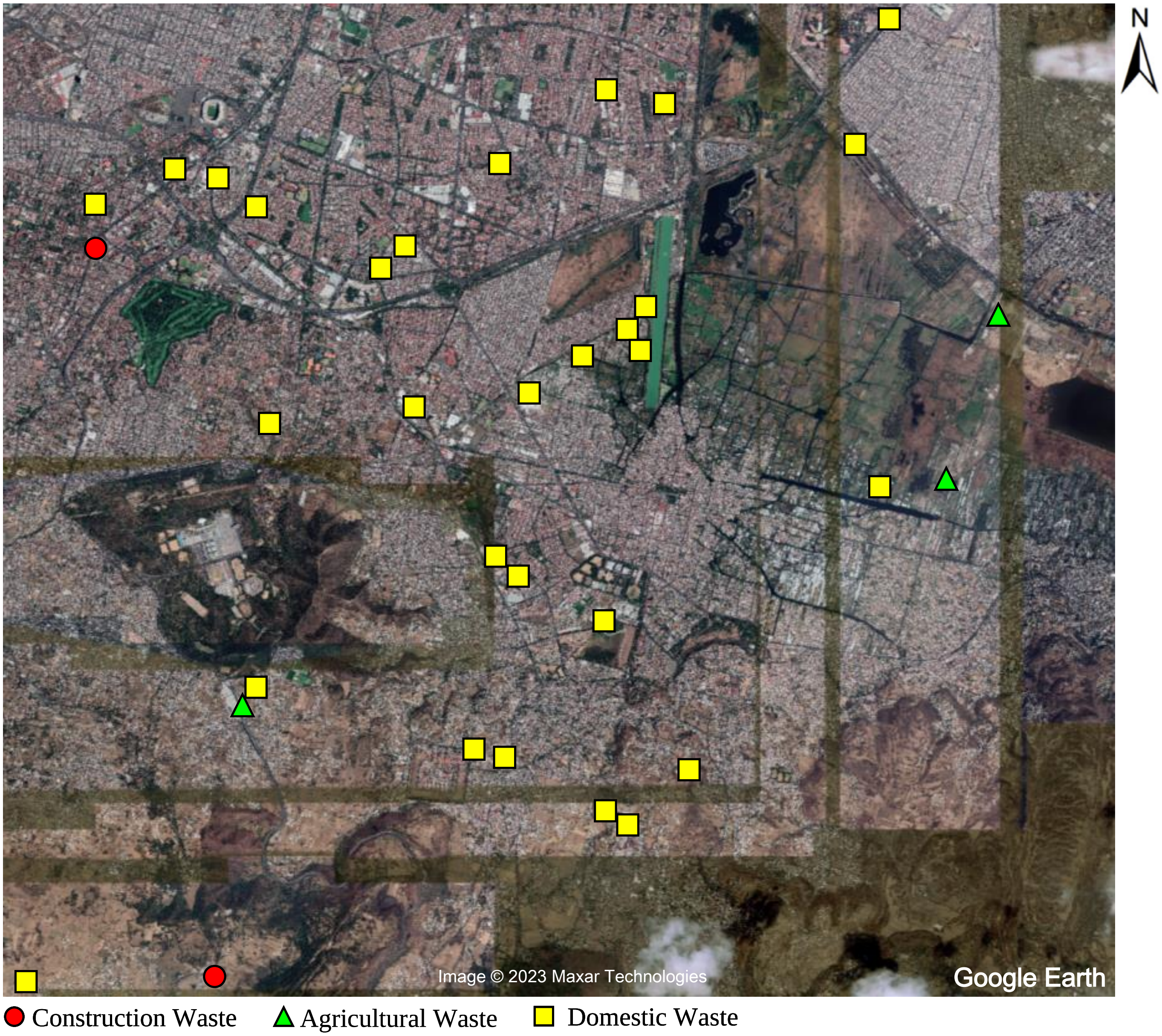

Supplementary Figure 11: Dumpsite distribution in parts of Mexicocity.

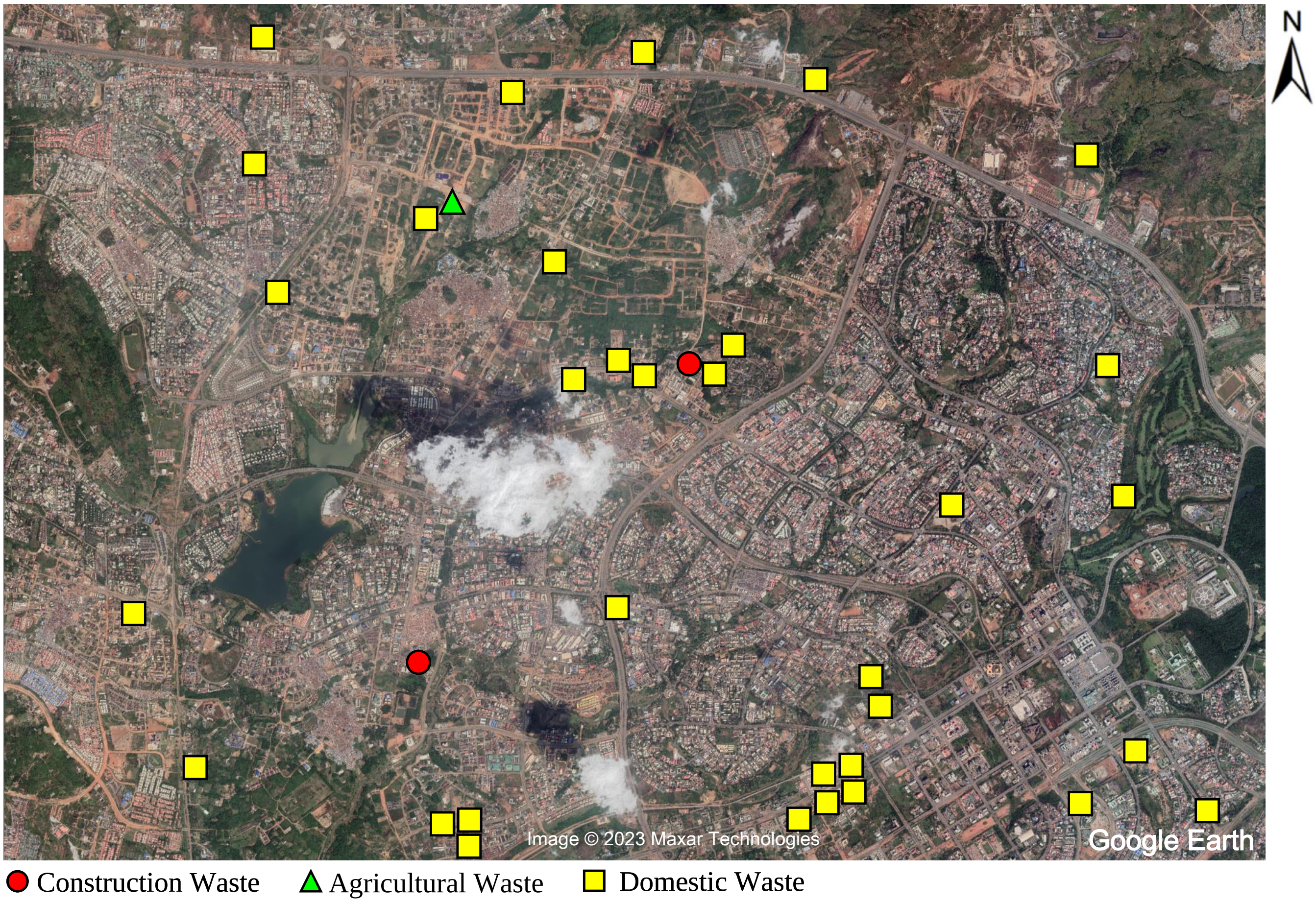

Supplementary Figure 12: Dumpsite distribution in parts of Winnipeg.

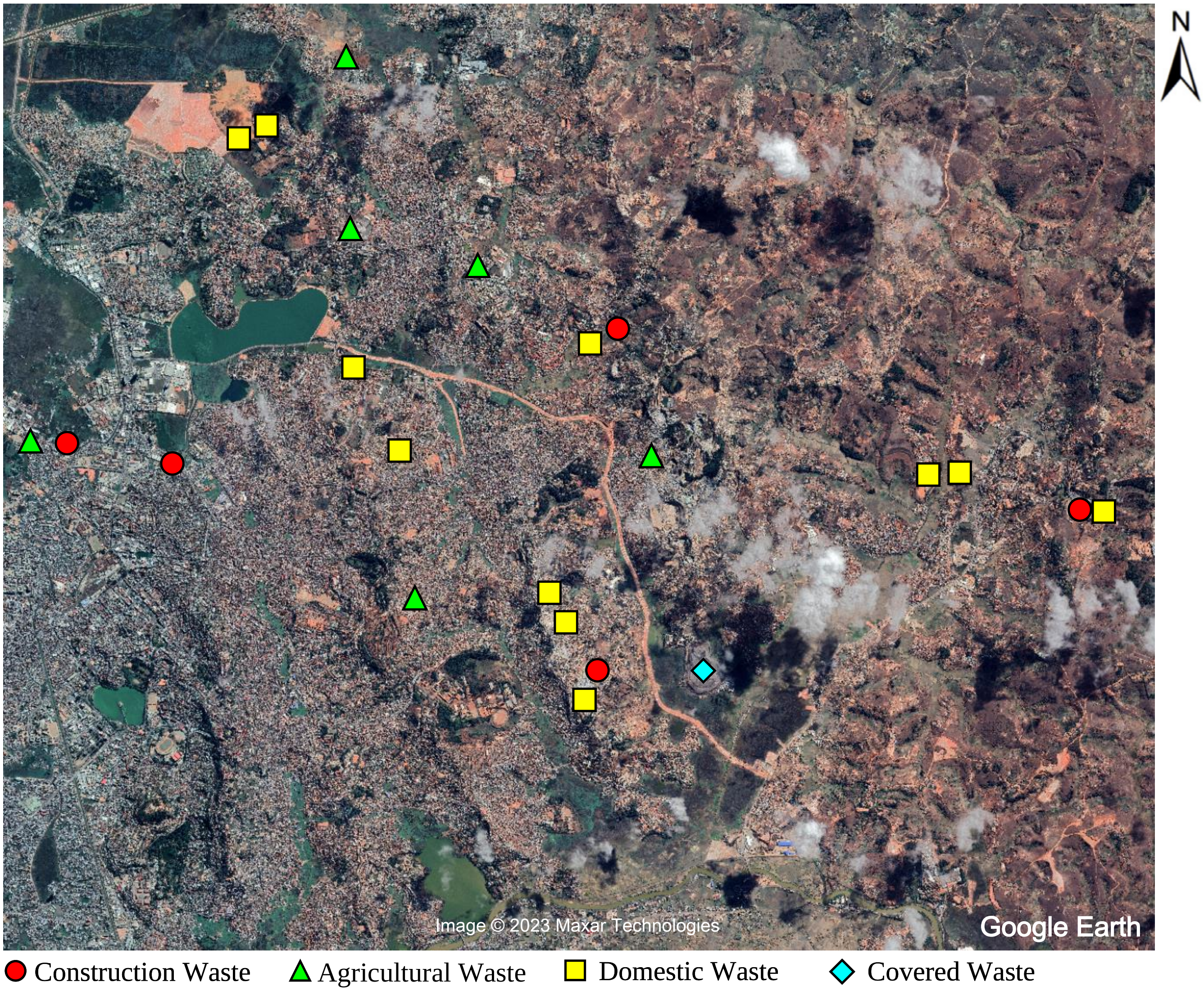

Supplementary Figure 13: Dumpsite distribution in parts of Antananarivo.

Introduction

This is a project for garbage dump detection with BCA-Net, which can be used to perform global garbage dump detection with our upcoming multi-category garbage dump dataset.

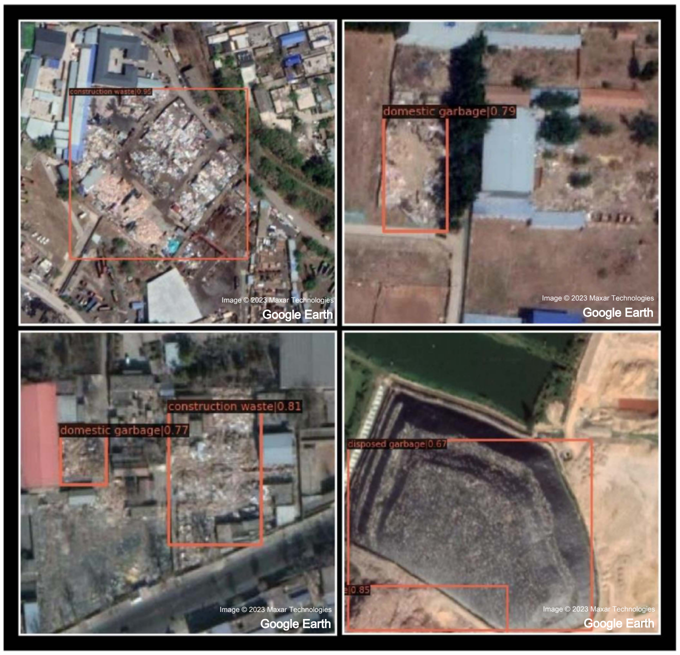

Prerequisites

- OS: Ubuntu 16.04
- GPU: Nvidia GTX/RTX series GPU with proper NVIDIA Driver installed
- Software: Docker installed

Installation

1. Create a new project folder.

```
mkdir /home/$[YOUR_USERNAME]/garbage_dump
cd /home/$[YOUR_USERNAME]/garbage_dump
```

2. Download the code.

```
git clone https://github.com/DongshuoYin/garbage_dump_detection.git
```

3. Download dataset in our paper's link.

4. Unzip the dataset to `./garbage_dump_detection/data/` .

```
garbage_dump_detection
├── mmdet
├── tools
├── configs
├── data
│   ├── garbage_dump_2022
│   │   ├── VOC2012
│   │   │   ├─train
│   │   │   │   ├─Annotations
│   │   │   │   ├─JPEGImages
│   │   │   │   ├─train.txt
│   │   │   ├─test
│   │   │   │   ├─Annotations
│   │   │   │   ├─JPEGImages
│   │   │   │   ├─test.txt
......
......
```

5. Get the docker image from Docker-hub.

```
sudo docker pull y389164605/garbage_dump_detection:latest
```

6. Create a docker container with the above image.

```
sudo nvidia-docker run --privileged=true --name=$[YOUR_CONTAINER_NAME] --shm-size=8g -d -p $[PORT_FOR_CON
```

Note:

a. If the terminal remains inactive, create a new terminal and continue the operation.

b. `/home/$[YOUR_USERNAME]/garbage_dump/garbage_dump_detection` in your computer and `/garbage_dump_detection` in your docker container are a pair of mapped folders and they will remain consistent.

7. Enter the above docker container.

```
sudo docker exec -it $[YOUR_CONTAINER_NAME] /bin/bash
cd /garbage_dump_detection
python setup.py develop
```

Demo

1. Download the pre-trained model here and put it in `/home/$[YOUR_USERNAME]/garbage_dump/garbage_dump_detection/checkpoint_backup/`

2. Run the following code in container.

```
cd tools
python demo.py
```

Batch inference

1. Download the pre-trained model here (same as Demo) and put it in `/home/$[YOUR_USERNAME]/garbage_dump/garbage_dump_detection/checkpoint_backup/` .

2. Resize your images to 1024*1024 pixels.

3. Copy all your images to `/home/$[YOUR_USERNAME]/garbage_dump/garbage_dump_detection/tools/batch_inference_data/` .

4. Run the following code in container.

```
cd tools
python inference.py
```

5. Check your imference results in `/home/$[YOUR_USERNAME]/garbage_dump/garbage_dump_detection/tools/batch_inference_data/inference_visualization`

Training the BCA-Net

1. Run the following code.

```
cd tools
python train.py ../_myconfigs/pascal_voc/faster_rcnn_r50_fpn_1x_voc0712_all_layer_SE_with_ClassBalancedDa
```

2. Check the evaluation metric on test set after each epoch.

Note: If you want to train with your own dataset, replace the dataset in `/home/$[YOUR_USERNAME]/garbage_dump/garbage_dump_detection/data` with yours and keep the data and folder format the same as ours.

Supplementary Figure 14: We have designed a detection tool that can be run directly without caring about the code.

Supplementary Tables

Supplementary Table 9: Number of dumpsites in 28 regions. "Better Developed" indicates whether the city is the better developed one in its country.

Country	City	Better Developed	DW	CW	AW	CW2	ALL
Brazil	Fortaleza	N	23	2	2	0	27
Brazil	Rio	Y	14	1	0	0	15
Canada	Winnipeg	N	33	2	2	0	37
Canada	Toronto	Y	8	0	2	0	10
China	Shantou	N	33	3	6	0	42
China	Shanghai	Y	9	2	5	0	16
Germany	Munich	Y	4	2	1	0	7
Germany	Leipzig	N	3	0	0	0	3
India	Lucknow	-	25	6	3	0	34
Japan	Nagano	N	16	5	5	0	26
Japan	Tokyo	Y	2	0	0	0	2
Latvia	Riga	Y	28	4	2	0	34
Latvia	Latgale	N	8	1	0	0	9
Madagascar	Antananarivo	Y	11	5	6	1	23
Madagascar	Toamasina	N	5	2	1	0	8
Mexico	Ecatepec	N	47	3	6	0	56
Mexico	Mexico City	Y	29	2	3	0	34
New Zealand	Napier	N	6	0	2	0	8
New Zealand	Auckland	Y	2	1	1	0	4
Nigeria	Lagos	-	32	9	1	0	42
Russia	Volgograd	N	27	1	0	0	28
Russia	Moscow	Y	7	1	0	0	8
South Africa	Bojanala	N	41	13	10	0	64
South Africa	Johannesburg	Y	51	3	8	1	63
Turkey	Gaziantep	N	35	5	6	0	66
Turkey	Istanbul	Y	18	2	3	0	23
Uganda	Kampala	Y	41	3	4	0	48
Uganda	Gulu	N	28	0	10	0	38

Supplementary Table 10: The values of seventeen attributes. The full abbreviations are as follows: URC: Urbanization Ranking by Country (CIA), III: International Innovation Index (Boston Consulting Group), GNI: Gross National Income (World Bank), DC or N: Developed Country or Not (World Population Review), WTRP: Waste Treatment Recycling Percent (World Bank), WTIP: Waste Treatment Incineration Percent (World Bank), WTODP: Waste Treatment Open Dump Percent (World Bank), TMSWGTY: Total Municipal Solid Waste Generated Tons Year (World Bank), GWI: Global Waste Index 2019 (Sensonseo), AL: Absolute Latitude, Latitude, NSH: Northern and Southern Hemisphere, GDPPC: GDP per capita (World Bank), NGDP: National GDP (United Nations), PD: Population Density, CCITW: Cleanest Countries In The World 2021 (World Population Review), ERBC: Education Rankings By Country 2020 (World Population Review).

Country	City	URC	III	GNI	DC or N (0-No, 1-Yes)	WTRP (%)	WTIP (%)	WTODP	TMSWGTY	GWI	AL	L	NSH	GDPPC (US$)	NGDP (million US$)	Population	PD (per km^2)	CCITW	ERBC
Brazil	Fortaleza	26	72	90	0	1.4		15.61932	79069584		3.73	-3.73	S	14,836	1,847,795	4,073,465	560	54	30
Brazil	Rio	26	72	90	0	1.4		15.61932	79069584		22.9	-22.9	S	14,836	1,847,795	13,458,075	2,705	54	30
Canada	Winnipeg	38	14	22	1	20.59	3		25103034	51.19	49.89	49.89	N	48,073	1,741,496	816,593	147	21	3
Canada	Toronto	38	14	22	1	20.59	3		25103034	51.19	43.65	43.65	N	48,073	1,741,496	6,196,731	4,334	21	3
China	Shantou	96	5	81	0		29.84	8.21	395081376		23.35	23.35	N	17,312	14,342,933	4,327,316	1,300	118	24
China	Shanghai	96	5	81	0		29.84	8.21	395081376		31.23	31.23	N	17,312	14,342,933	27,058,480	3,900	118	24
Germany	Munich	53	7	19	1	47.83	31.71		50627876	87.32	48.13	48.13	N	53,694	3,861,123	1,538,302	4,800	10	4
Germany	Leipzig	53	7	19	1	47.83	31.71		50627876	87.32	51.34	51.34	N	53,694	3,861,123	591,052	2,000	10	4
India	Lucknow	77	51	177	0	5		77	189750000		26.85	26.85	N	6,454	2,891,582	3,676,515	5,500	162	34
Japan	Nagano	15	1	29	1	4.9	80.2		42720000	92.48	36.15	36.15	N	42,197	5,082,465	370,632	440	12	11
Japan	Tokyo	15	1	29	1	4.9	80.2		42720000	92.48	35.67	35.67	N	42,197	5,082,465	37,393,128	6,363	12	11
Latvia	Riga	77	43	56	1	21.23			839714.375	11.63	56.92	56.92	N	32,019	34,102	630,692	147	36	50
Latvia	Latgale	77	43	56	1	21.23			839714.375	11.63	55.93	55.93	N	32,019	34,102	255,968	18	36	50
Madagascar	Antananarivo	148	103	235	0			96.7	3768759.16		18.87	-18.87	S	1,593	14,104	3,368,585	15,000	168	
Madagascar	Toamasina	148	103	235	0			96.7	3768759.16		18.14	-18.14	S	1,593	14,104	325,857	11,000	168	
Mexico	Ecatepec	44	57	89	0	5		21	53100000	32.15	19.6	19.6	N	18,833	1,256,440	1,645,352	10,534	50	36
Mexico	Mexico City	44	57	89	0	5		21	53100000	32.15	19.42	19.42	N	18,833	1,256,440	21,782,378	6,200	50	
New Zealand	Napier	28	26	26	1				3405000	24.37	39.51	-39.51	S	44,252	206,936	66,300	630	19	14
New Zealand	Auckland	28	26	26	1				3405000	24.37	36.85	-36.85	S	44,252	206,936	1,606,564	2,400	19	36
Nigeria	Lagos	124	88	178	0				27614830.07		6.52	6.52	N	5,187	474,516	14,368,332	6,871	146	14
Russia	Volgograd	59	49	77	0	4.5		95	60000000		48.7	48.7	N	28,213	1,692,930	1,004,688	1,200	57	21
Russia	Moscow	59	49	77	0	4.5		95	60000000		55.75	55.75	N	28,213	1,692,930	12,537,954	4,882	57	21
South Africa	Bojanala	81	34	121	0	28			18457232		25.38	-25.38	S	12,096	351,430	1,507,505	82	93	32
South Africa	Johannesburg	81	34	121	0	28			18457232		26.2	-26.2	S	12,096	351,430	5,782,747	3,400	93	32
Turkey	Gaziantep	56	58	85	0			44	35374156	0	37.06	37.06	N	28,120	761,425	1,704,118	580	97	31
Turkey	Istanbul	56	58	85	0			44	35374156	0	41.27	41.27	N	28,120	761,425	15,190,336	2,894	97	31
Uganda	Kampala	175	90	225	0	6		87	7045049.836		0.34	0.34	N	2,297	32,609	3,298,364	3,021	124	
Uganda	Gulu	175	90	225	0	6		87	7045049.836		2.77	2.77	N	2,297	32,609	152,276	115	124	

Supplementary Table 11: The spearman correlation analysis results of 17 attributes and the number of dumpsites. Each attribute has two rows of correlation indicators, where the first row is the correlation coefficient and the second row is the P-Value. P<0.05 indicates statistically linear correlation, which is indicated by *. P<0.01 indicates a strong correlation, which is represented by **. Other correlations are not statistically relevant.

Attributes	Parameter	All	Domestic Waste	Construction Waste	Agricultural Waste
Urbanization Ranking by Country	Correlation coefficient	**0.418***	0.351	0.37	**0.381***
	P-Value	0.027	0.067	0.053	0.046
International Innovation Index	Correlation coefficient	0.311	0.294	0.222	0.124
	P-Value	0.108	0.128	0.257	0.528
Gross National Income	Correlation coefficient	**0.538****	**0.480****	**0.486****	**0.455***
	P-Value	0.003	0.010	0.009	0.015
Developed Country or Not(0-No, 1-Yes)	Correlation coefficient	**-0.554****	**-0.536****	**-0.403***	**0.420***
	P-Value	0.002	0.003	0.034	0.026
Waste Treatment Recycling Percent	Correlation coefficient	0.133	0.109	0.117	0.218
	P-Value	0.586	0.655	0.634	0.370
Waste Treatment Incineration Percent	Correlation coefficient	-0.488	-0.515	0.103	-0.323
	P-Value	0.220	0.191	0.809	0.435
Waste Treatment Open Dump Percent	Correlation coefficient	-0.214	-0.273	-0.044	-0.161
	P-Value	0.445	0.325	0.875	0.567
Total Municipal Solid Waste Generated Tons Year	Correlation coefficient	0.036	0.071	0.037	-0.053
	P-Value	0.856	0.718	0.853	0.79
Global Waste Index 2019	Correlation coefficient	-0.381	-0.395	-0.254	-0.298
	P-Value	0.178	0.162	0.380	0.301
Absolute Latitude	Correlation coefficient	**-0.424***	-0.365	-0.312	**-0.463***
	P-Value	0.024	0.056	0.107	0.013
Latitude	Correlation coefficient	-0.204	-0.144	-0.149	**-0.377***
	P-Value	0.299	0.466	0.450	0.048
Northern and Southern Hemisphere	Correlation coefficient	-0.083	-0.118	0.025	0.074
	P-Value	0.673	0.551	0.900	0.707
GDP per capita (US$ World Bank)	Correlation coefficient	-0.356	-0.353	-0.230	**-0.455***
	P-Value	0.063	0.066	0.240	0.015
National GDP (million US$ United Nations)	Correlation coefficient	0.096	0.049	0.269	0.187
	P-Value	0.626	0.806	0.166	0.340
Population	Correlation coefficient	-0.099	-0.073	-0.129	0.032
	P-Value	0.616	0.712	0.512	0.873
Population Density (/km^2)	Correlation coefficient	-0.188	-0.188	0.090	-0.108
	P-Value	0.337	0.339	0.650	0.584S
Cleanest Countries In The World 2021	Correlation coefficient	**0.487****	**0.424***	**0.475***	**0.433***
	P-Value	0.009	0.025	0.011	0.021
Education Rankings By Country 2020	Correlation coefficient	0.366	0.351	0.382	0.294
	P-Value	0.086	0.101	0.072	0.174

References

[1] Shaoqing Ren, Kaiming He, Ross Girshick, and Jian Sun. Faster r-cnn: Towards real-time object detection with region proposal networks. *Advances in neural information processing systems*, 28:91–99, 2015.

[2] Kaiming He, Xiangyu Zhang, Shaoqing Ren, and Jian Sun. Deep residual learning for image recognition. In *Proceedings of the IEEE conference on computer vision and pattern recognition*, pages 770–778, 2016.

[3] Tsung-Yi Lin, Piotr Dollár, Ross Girshick, Kaiming He, Bharath Hariharan, and Serge Belongie. Feature pyramid networks for object detection. In *Proceedings of the IEEE conference on computer vision and pattern recognition*, pages 2117–2125, 2017.

[4] Ashish Vaswani, Noam Shazeer, Niki Parmar, Jakob Uszkoreit, Llion Jones, Aidan N Gomez, Łukasz Kaiser, and Illia Polosukhin. Attention is all you need. In *Advances in neural information processing systems*, pages 5998–6008, 2017.

[5] Jie Hu, Li Shen, and Gang Sun. Squeeze-and-excitation networks. In *Proceedings of the IEEE conference on computer vision and pattern recognition*, pages 7132–7141, 2018.

[6] Sanghyun Woo, Jongchan Park, Joon-Young Lee, and In So Kweon. Cbam: Convolutional block attention module. In *Proceedings of the European conference on computer vision (ECCV)*, pages 3–19, 2018.

[7] Jyoti K Chetri and Krishna R Reddy. Advancements in municipal solid waste landfill cover system: A review. *Journal of the Indian Institute of Science*, 101(4):557–588, 2021.

[8] Jun Jiang, Jianhua Li, and Sami Rtimi. Investigation and modeling of odors release from membrane holes on daily overlay in a landfill and its impact on landfill odor control. *Environmental Science and Pollution Research*, 28(4):4443–4451, 2021.

[9] Ying Wang, Lin Li, Zhongping Qiu, Kaixiong Yang, Yunping Han, Fengguang Chai, Pengyu Li, and Yanjie Wang. Trace volatile compounds in the air of domestic waste landfill site: Identification, olfactory effect and cancer risk. *Chemosphere*, 272:129582, 2021.

[10] Yuansheng Hua, Lichao Mou, Pu Jin, and Xiao Xiang Zhu. Multiscene: A large-scale dataset and benchmark for multiscene recognition in single aerial images. *IEEE Transactions on Geoscience and Remote Sensing*, 60:1–13, 2021.

[11] M Schmitt and Y-L Wu. Remote sensing image classification with the sen12ms dataset. *ISPRS Annals of Photogrammetry, Remote Sensing and Spatial Information Sciences*, 52:101–106, 2021.

[12] Kaihua Tang, Jianqiang Huang, and Hanwang Zhang. Long-tailed classification by keeping the good and removing the bad momentum causal effect. *Advances in Neural Information Processing Systems*, 33, 2020.

[13] Harsh Panwar, PK Gupta, Mohammad Khubeb Siddiqui, Ruben Morales-Menendez, Prakhar Bhardwaj, Sudhansh Sharma, and Iqbal H Sarker. Aquavision: Automating the detection of waste in water bodies using deep transfer learning. *Case Studies in Chemical and Environmental Engineering*, 2:100026, 2020.

[14] Riadh Ayachi, Mouna Afif, Yahia Said, and Mohamed Atri. Traffic signs detection for real-world application of an advanced driving assisting system using deep learning. *Neural Processing Letters*, 51(1):837–851, 2020.

[15] Xian Sun, Yingfei Liu, Zhiyuan Yan, Peijin Wang, Wenhui Diao, and Kun Fu. Sraf-net: Shape robust anchor-free network for garbage dumps in remote sensing imagery. *IEEE Transactions on Geoscience and Remote Sensing*, 2020.

[16] Tsung-Yi Lin, Priya Goyal, Ross Girshick, Kaiming He, and Piotr Dollár. Focal loss for dense object detection. In *Proceedings of the IEEE international conference on computer vision*, pages 2980–2988, 2017.

[17] Rocio Nahime Torres and Piero Fraternali. Learning to identify illegal landfills through scene classification in aerial images. *Remote Sensing*, 13(22):4520, 2021.

[18] Rocio Nahime Torres, Piero Fraternali, and Andrea Biscontini. On the use of class activation maps in remote sensing: the case of illegal landfills. In *2021 IEEE 8th International Conference on Data Science and Advanced Analytics (DSAA)*, pages 1–10. IEEE, 2021.

[19] Shynggys Abdukhamet. *Landfill Detection in Satellite Images Using Deep Learning.* PhD thesis, Shanghai Jiao Tong University Shanghai, China, 2019.

[20] Rocio Nahime Torres, Piero Fraternali, and Jesus Romero. Odin: an object detection and instance segmentation diagnosis framework. In *European Conference on Computer Vision*, pages 19–31. Springer, 2020.

[21] Ramprasaath R Selvaraju, Michael Cogswell, Abhishek Das, Ramakrishna Vedantam, Devi Parikh, and Dhruv Batra. Grad-cam: Visual explanations from deep networks via gradient-based localization. In *Proceedings of the IEEE international conference on computer vision*, pages 618–626, 2017.

[22] China centre for resources satellite data and application. `https://www.cresda.com/zgzywxyyzx/index.html`.

[23] Spacewill. `http://en.spacewillinfo.com/`.

[24] Lungile Selani. *Mapping Illegal Dumping Using a High Resolution Remote Sensing Image Case Study: Soweto Township in South Africa.* PhD thesis, University of the Witwatersrand, Faculty of Science, School of Geography . . . , 2017.

[25] Mónica Duque-Acevedo, Luis J Belmonte-Urena, Francisco Joaquín Cortés-García, and Francisco Camacho-Ferre. Agricultural waste: Review of the evolution, approaches and perspectives on alternative uses. *Global Ecology and Conservation*, 22:e00902, 2020.

[26] FO Obi, BO Ugwuishiwu, and JN Nwakaire. Agricultural waste concept, generation, utilization and management. *Nigerian Journal of Technology*, 35(4):957–964, 2016.

[27] Chrysanthos Maraveas. Production of sustainable and biodegradable polymers from agricultural waste. *Polymers*, 12(5):1127, 2020.
